# Controlling Multiphase Coacervate Wetting and Self-Organization by Interfacial Proteins

**DOI:** 10.1021/jacs.5c03870

**Published:** 2025-06-17

**Authors:** Tiemei Lu, Susanne Liese, Brent S. Visser, Merlijn H. I. van Haren, Wojciech P. Lipiński, Wilhelm T. S. Huck, Christoph A. Weber, Evan Spruijt

**Affiliations:** † Institute for Molecules and Materials, 6029 Radboud University, Heyendaalseweg 135, Nijmegen 6525 AJ, The Netherlands; ‡ Department of Chemistry, University of Oxford, 12 Mansfield Road, Oxford OX1 3TA, U.K.; § Faculty of Mathematics, Natural Science, and Materials Engineering, Institute of Physics, 26522 University of Augsburg, Universitätsstr. 1, Augsburg 86159, Germany

## Abstract

Biomolecular condensates help organize biochemical processes in cells and synthetic cell analogues. Many condensates exhibit multiphase architectures, yielding compartments with distinct functions. However, how cells regulate the transformation between different multiphase architectures remains poorly understood. Here, we use multiphase coacervates as model condensates and present a new approach to control wetting and self-organization in multiphase coacervates by introducing a surface-active protein, α-synuclein (αSyn). αSyn can localize at the interface of uridine 5′-triphosphate (UTP)/poly-l-lysine (pLL)/oligo-l-arginine (R_10_) multiphase coacervates and induce the transformation from nested droplets into partially wetted droplets. The exposed UTP/R_10_ core coacervate droplets adhered to neighboring (shell) coacervates, forming structures similar to polymers and leading to a dynamic yet stable self-organized network of connected coacervates, which we call coacervate polymers. A theoretical model demonstrates that multiphase coacervates transition to partial wetting upon increasing the interfacial protein, consistent with experimental observations. When three neighboring coacervates are not aligned, surface tension straightens their arrangement, similar to semiflexible polymers. This mechanism likely extends to larger structures, promoting chain formation while preventing fusion. Interestingly, diverse proteins were found to be surface active in multiphase coacervates: BSA, mCherry, and FtsZ all exhibited the same effect on multiphase coacervates’ partial wetting and organization. These findings suggest that interfacial proteins could be used by cells not only to stabilize condensates, but also to control multiphase organization and to regulate the interaction between condensates.

## Graphical abstract

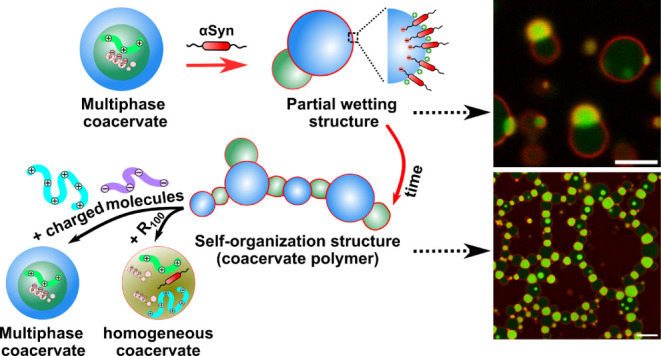

## Introduction

Biomolecular condensates are liquid-like bodies formed by liquid–liquid phase separation (LLPS) that play an important role in cellular organization.^1^ Coacervate droplets are frequently used as condensate models to study the impact of condensates on, for instance, chemical reactions, enzyme activity and protein aggregation.^2^ Recent studies indicate that biomolecular condensate architectures in cellular media are often more complex than a simple liquid droplets: interfacial phenomena play an important role in controlling condensate size, shape and interactions,^3^ and many condensates have multiphase or multidomain architectures with potentially distinct functions.^4^ The multiphase architectures of condensates include core–shell architectures, partially wetted structures, and condensates with multiple coexisting internal phases, which can vary depending on the cell cycle phase, cell differentiation or stress response.^5^ However, how cells regulate the transformation between different multiphase architectures remains poorly understood.^6,7^

The formation and stability of multiphase droplets have been studied in detail using coacervate model systems. Multiphase coacervates with droplet-in-droplet architectures, multiple subcompartments and up to three coexisting layers have been reported.^8^ In almost all these examples, complete engulfment of one type of coacervate by another is observed. To dynamically control multiphase coacervate morphology, studies show that adjusting the composition or biopolymer length in multiphase coacervates can drive morphological transitions from fully engulfed to partially engulfed, and ultimately to nonengulfing droplets.^9,10^ However, whether cells could dynamically control multiphase condensate architecture without changing their composition is not clear. To shift between different multiphase architectures requires changing the interfacial tensions between the different condensate phases. Proteins that localize to the interface of condensates may be able to transform multiphase droplets to demixed and separated droplets. Various studies have shown that proteins, protein clusters and protein filaments can localize at condensate interfaces and stabilize them.^11,12^ These findings suggest that interfacial proteins can modulate the coacervate surface energy. We therefore hypothesized that interfacial localization of proteins can also be used to induce transformation of multiphase coacervate architectures.

To test our hypothesis, we studied the effect of the disordered amyloidogenic protein α-synuclein (αSyn) on multiphase coacervates. αSyn has recently been reported to localize to coacervate interfaces.^12,13^ We found that αSyn can also localize at the interface of uridine 5′-triphosphate (UTP)/ poly-l-lysine (pLL)/ oligo-l-arginine (R_10_) multiphase coacervates and induce their transformation from a nested architecture into a partially wetted touching droplet architecture, accompanied by the accumulation of αSyn around the shell (UTP/pLL) and core (UTP/R_10_) coacervate phases. Interestingly, the exposed UTP/R_10_ core coacervate droplets could adhere to neighboring (shell) coacervates, acting as a glue and linking shell and core coacervates together, facilitating their self-organization into higher-order, interconnected, chain-like structures, similar to previously reported chain-like structures based on surfactants and synthetic polyelectrolytes.^14^ In this case, the use of an interfacial protein in the chain-like structures allows for dynamic and biochemical control over the formation and connectivity. We demonstrate that interfacial proteins provide a general strategy for controlling multiphase coacervate architectures: besides αSyn, several other globular and disordered proteins with negatively charged domains (BSA, mCherry, and FtsZ) are able to induce transformation from nested droplets to partially wetted structures, and further self-organization into chain-like structures. Our findings suggest that interfacial proteins could be used by cells not only to stabilize condensates, but also to control multiphase architectures and to regulate the interaction between condensates. The reversibility of the transitions we observed opens the way to utilize this mechanism in the context of synthetic cells and tissues to control protocell-protocell interactions and communication.

## Experimental Section

### Materials

All chemicals and reagents were used as received from commercial suppliers unless stated otherwise. Milli-Q water (MQ, 18.2 MΩ·cm) from Millipore Corporation was used. The following chemicals were purchased from Sigma-Aldrich: poly-l-lysine hydrobromide (pLL, 15–30 kDa), polyadenylic acid potassium salt (polyA), bovine serum albumin labeled with tetramethylrhodamine isothiocyanate (BSA-TRITC), adenosine 5′-triphosphate disodium salt hydrate (ATP), uridine 5′-triphosphate trisodium salt hydrate (UTP), hexadecyltrimethylammonium bromide (CTAB), sodium chloride (NaCl), magnesium chloride hexahydrate (MgCl_2_•6H_2_O), 4-(2-hydroxyethyl)-1-piperazineethanesulfonic acid (HEPES), and tris­(hydroxymethyl)-aminomethane hydrochloride (Tris-HCl). The following oligopeptides were purchased from Alamanda Polymers: poly­(oligo)-l-arginine hydrochloride (10-mer, R_10_, Mw = 1.9 kDa; 100-mer, R_100_, Mw = 19 kDa), poly-l-aspartic acid sodium salt (100-mer, D_100_, Mw = 14 kDa), methoxy-poly­(ethylene glycol)-*block*-poly­(l-glutamic acid sodium salt) (mPEG_1k_-*b*-pLE_100_ (PEG_1k_-*b*-pGlu_100_), pLE_100_, Mw = 15 kDa), methoxy-poly­(ethylene glycol)-*block*-poly­(l-lysine hydrochloride) (mPEG_5K_-*b*-pLL_100_, pLL_100_, Mw = 16 kDa), the polydispersity index (PDI) of all peptides was 1.0 to 1.2 and the purity was >90%. FAM-labeled R_10_ (FAM-R_10_, 3.1 kDa, which contains a 6-aminohexanoic acid between the peptide and FAM) was purchased from Genscript. mCherry (26.7 kDa), was purchased from OriGene (purity >80%). Filamenting temperature-sensitive mutant Z was expressed, purified and labeled (FtsZ-Alexa Fluor 647) as previously described.^15^ eGFP (84.7 μM) was produced and purified using a custom-made IVTT protocol as described elsewhere.^200^

### αSyn Protein Preparation and Labeling

Wild-type FL-αSyn, αSyn_1–108_, and the cysteine mutants (S9C) were expressed and purified as previously described.^16^ Purified proteins were stored at a concentration of ∼ 200 μM in 10 mM Tris-HCl (pH = 7.40) at – 80 °C, supplemented with 1 mM dithiothreitol (DTT) for the cysteine mutants. The cysteine mutants were labeled with Alexa Fluor 647 maleimide according to the dye manufacturer procedures.

### Coacervates Preparation and Chain-Like Structure Formation

Single-phase complex coacervates were prepared by first mixing Milli-Q water, NaCl (3.0 M stock), HEPES (0.50 M stock, pH = 7.40), MgCl_2_ (50 mM stock), and the negatively charged UTP in a microcentrifuge tube (0.5 mL, Eppendorf) at the required concentration, followed by the addition of positively charged R_10_, or pLL from their respective stock solutions in a 1:1 molar (monomer basis) ratio to the negatively charged UTP. The total volume of the mixtures was 20 μL. The final concentration of NaCl is 0 to 100 mM, and the final concentration of HEPES and MgCl_2_ are 50 mM, and 5 mM, respectively. Mixing was done by gentle pipetting (3×).

To prepare multiphase coacervates, two different types of complex coacervates were prepared separately, as described above, and then mixed together in a separate Eppendorf tube. Typically, in this paper, the ratio of multiphase coacervates UTP/pLL/R_10_ is 2:1:1 (UTP:pLL = 1:1, UTP:R_10_ = 1:1), and the concentrations of NaCl, HEPES, and Mg^2+^ are fixed at 30 mM, 50 mM, and 5 mM, respectively.

For chain-like structure formation, we used three methods. In the first method (the main method used in this paper), multiphase coacervates were prepared as described above, and then the charged proteins (αSyn, BSA-TRITC, mCherry, and FtsZ) or block copolymer (mPEG_1k_-*b*-pGlu_100_) were added, respectively. In the second method, αSyn-AF647 was added separately into the two types of complex coacervates, which were then mixed together. In the third method, αSyn-AF647 was added to one of the two types of droplets (UTP/R_10_), and then the two types of coacervate droplets were mixed together. All three methods yielded chain-like structures.

**Figure 1:**
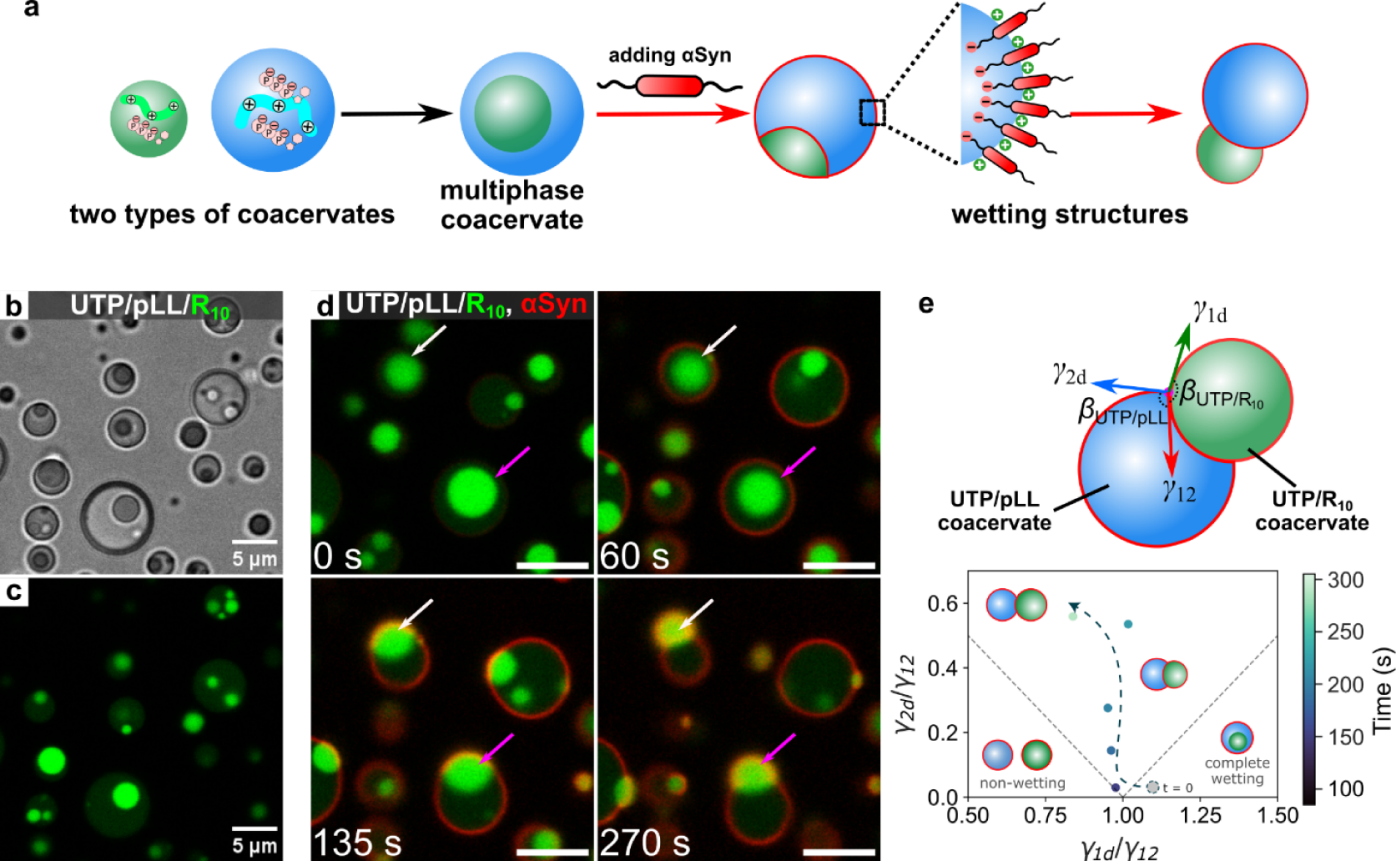
αSyn induces a wetting transition in multiphase coacervates. (a) Scheme of the formation of multiphase coacervates and partial wetting morphologies after adding Alexa Fluor 647 (AF647) labeled αSyn. (b,c) Multiphase coacervates of UTP/R_10_ (labeled with FAM-R_10_) core coacervates within a UTP/pLL shell coacervate phase, viewed in (b) bright-field and (c) confocal fluorescence microscopy with fluorescence from FAM-R_10_ (green), which is present in both phases. (d) Snapshots from confocal fluorescence microscopy illustrating the process of αSyn-induced partial wetting of UTP/pLL/R_10_ multiphase coacervates, with fluorescence from AF647-labeled αSyn (red) and FAM-R_10_ (green). The white and magenta arrows indicate the process of the core phase moving out of the shell phase. (e) Schematic illustration of vectors related to interfacial tensions (γ_1d_, γ_2d_, and γ_12_, “d”: dilute phase) and contact angles (β_UTP/pLL_ and β_UTP/R10_), along with a stability diagram showing the ratio of interfacial tensions over time following the addition of αSyn (Figure 1d). Experimentally, surface tensions were obtained by contact angle measurements as detailed in the Methods section. The dashed line is a guide to the eye. All scale bars represent 5 μm.

**Figure 2:**
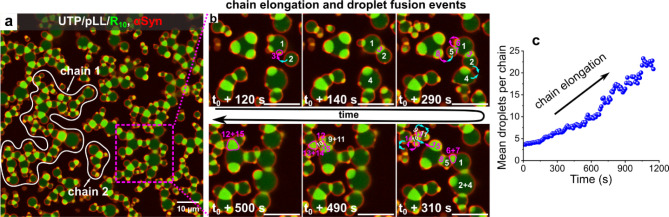
Self-organization of coacervates. (a) Composite confocal fluorescence image of UTP/pLL/R_10_ multiphase coacervates after the addition of αSyn at 1150s, showing chain-/network-like structures. Fluorescence from AF647-labeled αSyn (red) and FAM-R_10_ (green) is visible. (b) Snapshots from confocal fluorescence microscopy, zoomed in on the magenta dotted square in (a), illustrate the αSyn-induced self-organization process of the UTP/pLL/R_10_ multiphase coacervates. (c) Time-dependent plots show an increase in the average number of droplets per chain over time (chain elongation). Scale bars in (b) represent 5 μm.

**Figure 3:**
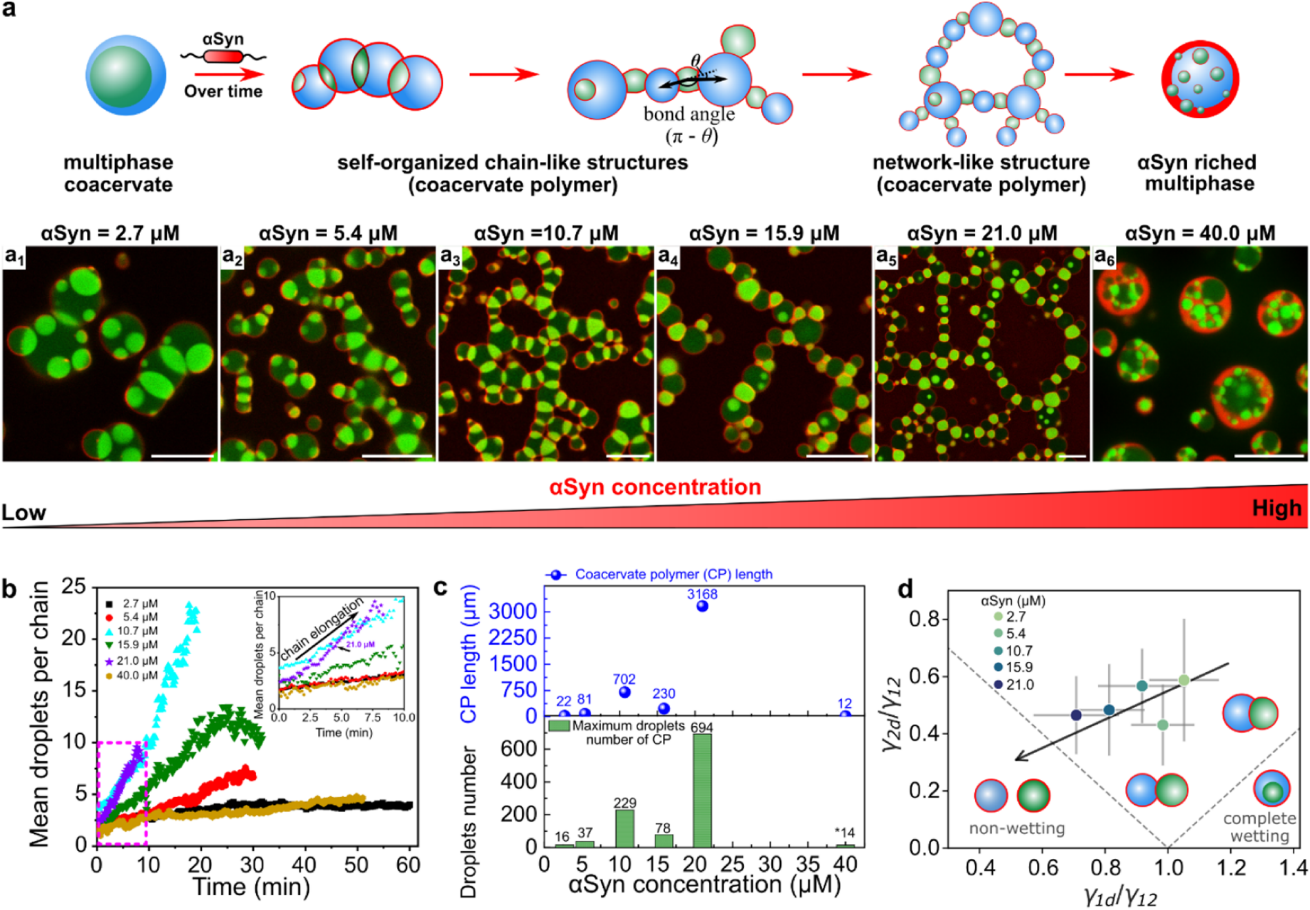
The concentration of αSyn impacts the self-organization. (a) Scheme of the self-organization after adding AF647 labeled αSyn and (a1–6) composite images showing diverse self-organized structures formed by adding different concentrations of αSyn to multiphase coacervates of UTP/pLL/R_10_: (a1) 2.7 μM, at 3600 s; (a2) 5.4 μM, at 1460 s; (a3) 10.7 μM, at 1260 s; (a4) 15.9 μM, at 1270 s; (a5) 21.0 μM, at 4080 s; and (a6) 40.0 μM, at 3000 s. All scale bars represent 10 μm. (b) Time-dependent plots show the average number of droplets per chain over time (chain elongation). The inset is zoomed in from the magenta square dotted line. (c) The maximum number of droplets of the self-organization structure (coacervate “polymer”) and their size (length) at different αSyn concentrations. (d) The stability diagram showing the average interfacial tension ratio varies in self-organized structures at different αSyn concentrations. For details on the determination of surface tensions, see Methods section.

**Figure 4:**
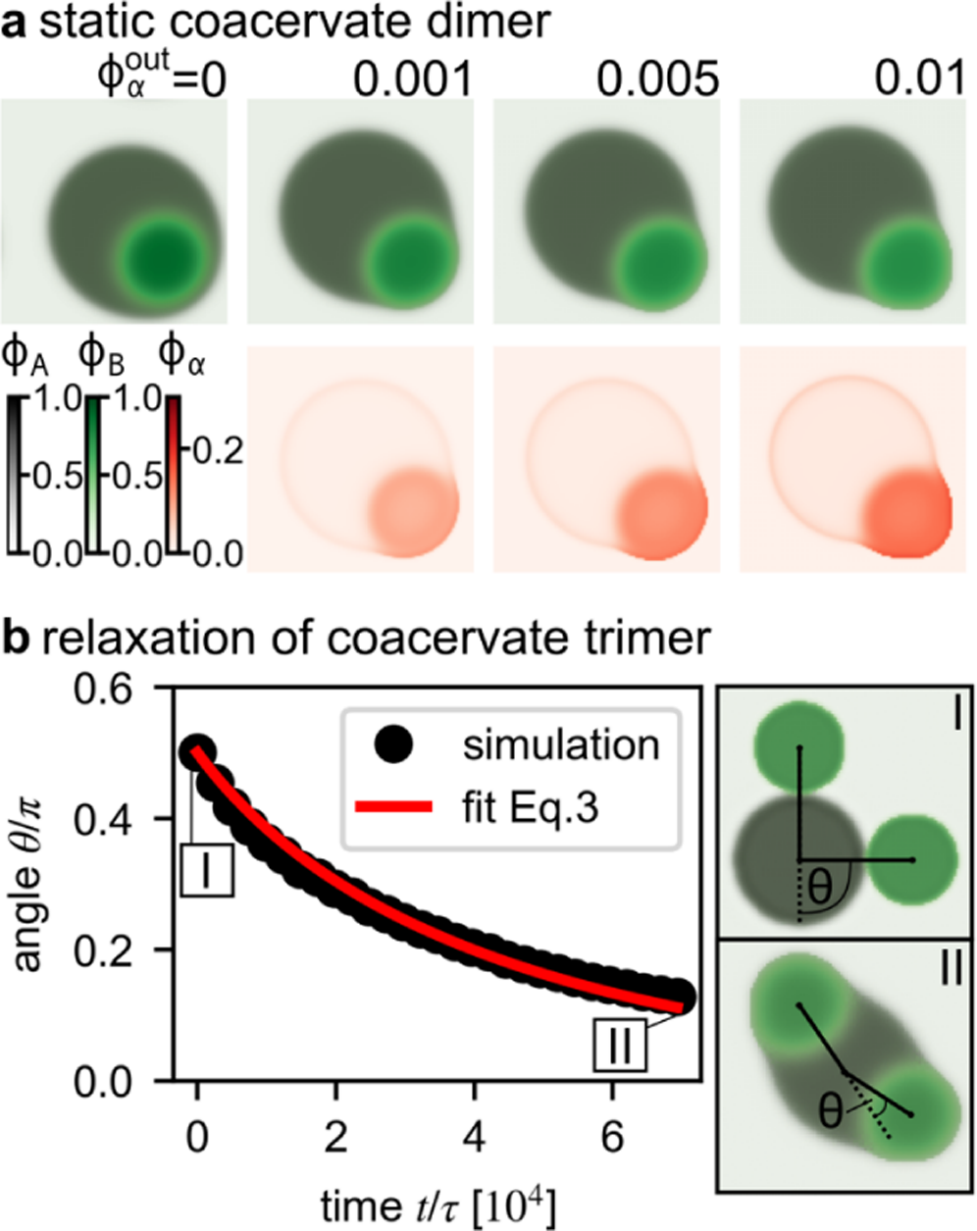
Angle dynamics of the multiphase coacervates. (a) Shape of a single coacervate dimer. The volume fraction $\phi_{\alpha }^{\left(out\right)}$ in the outer, dilute phase is varied. The volume fraction of the A and B components are shown as stacked colormaps for the stationary shape. The corresponding volume fraction of the α component is shown in the lower row. We see that increasing the interfacial component α triggers dimer formation and enhances dimer polarity. (b) Time evolution of the angle between three neighboring coacervates. A bent coacervate trimer with an initial angle θ=π/2 relaxes to a straight arrangement. The initial (I) and final (II) states of the simulations are shown on the right. This relaxation is reminiscent of an extensible semiflexible polymer with an effective bending rigidity and spring constant mediated by surface tensions and partial wetting. Time is rescaled by $\tau =\nu^{2/3}/\left(\Lambda^{\left(0\right)}k_{B}T\right)$ . By fitting, we find a relaxation time $\tau_{\mathrm{rel}}=1.3\times 10^{4}\tau$ .

**Figure 5:**
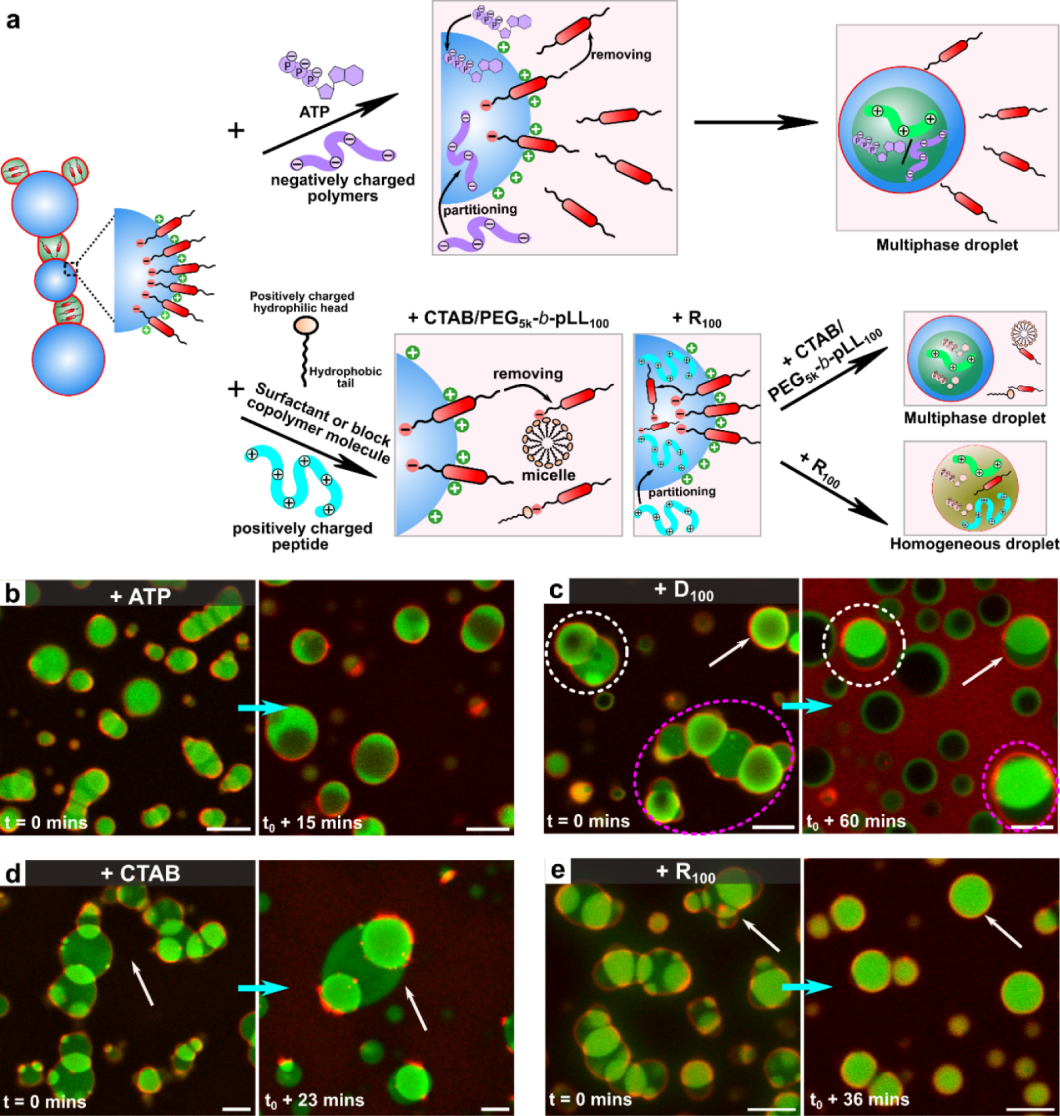
Control of self-organized multiphase coacervates by adding charged components. (a) A schematic illustrating structural changes upon the addition of negatively charged molecules or positively charged surfactant, block copolymer, and peptide into the chain-like structure. (b-e) Composite confocal images showing the transformation of the chain-like structure of UTP/pLL/R_10_ with αSyn after adding various charged molecules: (b,c) negatively charged (ATP, D_100_), and (d,e) positively charged (CTAB, R_100_). Images were recorded immediately after the addition of charged molecules (*t* = 0) and at relative steady state following complete interaction (final state time varies). Fluorescence is shown from FAM-R_10_ (green) and AF647-labeled αSyn (red). All scale bars represent 5 μm.

### Preparation of Modified μ-Slides

Samples were imaged in μ-slides with 8 wells, 18 wells, and 6 channels (No. 1.5, polymer coverslip, Ibidi GmbH). All slides used for microscopy were modified to minimize spreading of the coacervates on the surface of the slide. The surface intended to be modified was cleaned with oxygen plasma, and a solution (0.1 mg/mL) of PLL(20)-g[3.5]-PEG(2) (SuSoS, Dübendorf, Switzerland) dissolved in 10 mM HEPES buffer (pH = 7.40) was applied on the wells or channels of the μ-slides immediately after the plasma treatment. μ-slides were incubated with the PLL-*g*-PEG solution for 24 h at room temperature. Subsequently, slides were rinsed three times with Milli-Q water and dried with compressed N_2_. Modified slides were stored at room temperature.

### Confocal Microscopy Experiments

Images were obtained using either a Leica Liachroic Sp8 confocal microscope or a Leica TCS Sp8X confocal microscope. The Sp8 system was equipped with an EL6000 light source, a DFC7000 GT camera, a DMi8 CS motorized stage, LAS X SP8 controller software. A HC PL APO CS2 63×/1.40 oil objective was used. The Sp8X system was equipped with HyD and PMT detectors, and a pulsed white light laser. A HC PL APO CS2 100×/1.4 oil objective was used. Samples were visualized at an excitation of 488 nm (FAM-R_10_), 552 nm (mCherry, BSA-TRITC), and 649 nm (AF647 αSyn).

For microscopy experiments, samples were prepared in Eppendorf tubes. Typically, 10–30 μL of freshly prepared coacervate dispersion was directly added to a modified 18-well μ-slide chamber for imaging or video recording.

To study dynamic interactions between multiphase coacervates and negatively charged proteins or block copolymer, the coacervates were first added to a modified 6× μ-slide channel. The slide was placed on the microscope, and proteins or block copolymers were then introduced from one side of the channel. For competitive interfacial adsorption and salt stability tests, multiphase coacervates were first mixed with αSyn, added to the μ-slide channel, and incubated on the microscope for 20 to 30 min. Then, negatively or positively charged molecules or 3 M NaCl were added from the opposite side of the channel.

For the stability test, multiphase coacervates were mixed with charged proteins in a volume of 80 μL and added to a modified 8-well μ-slide chamber. To prevent evaporation, Milli-Q water was added to the empty wells to maintain a humid environment, and the slide cover was sealed with glue. After allowing the glue to dry for 10 to 15 min at room temperature, the slide was placed on the confocal microscope for imaging and video recording.

The partitioning coefficient (*K*_p_) of αSyn in coacervate droplets was determined from average fluorescence intensities using the equation: *K*_p_ = *I*_coa._/*I*_d_, where *I*_coa._, and *I*_d_ are the intensities inside of a coacervate, and in the dilute phase surrounding the coacervate droplets, respectively.

To quantify the enrichment of αSyn (or BSA-TRITC and mCherry) at the UTP/pLL shell interface and the core–shell interface, an interfacial partition coefficient (*K*_p_int_) was calculated. Several line profiles were drawn from the droplet center to the surrounding medium, and the maximum fluorescence intensity along each line was recorded. This process was repeated for 5–10 droplets, and the average maximum intensity was defined as *I*_int_. The interfacial partition coefficient was then calculated as *K*_p_int_ = *I*_int_/*I*_d_, where *I*_d_ is the average fluorescence intensity along the radial line within the dilute phase.

The accumulated concentration (*C*_accum_) of interfacial proteins at the droplet interface or within the coacervate phase was estimated using the relationship: *C*_accum_ = *K*_p_ × *C*_tot_. Assuming equilibrium partitioning and that the concentration in the dilute phase (*C*_dil_) is approximately equal to the total added concentration (*C*_tot_), this simplifies to *C*_accum_ = *K*_p_ × *C*_dil_ ≈ *K*_p_ × *C*_tot_.

### Wide-Field Microscopy

Images for coacervates formed by negatively charged molecules (UTP, ATP, D_100_, and polyA) with either pLL or R_10_, as well as UTP with R_100_ were obtained using an epi-fluorescent microscope (Leica DMi8), equipped with Sola LED and white LED light sources, a DFC7000 T camera, and a 100×/1.44 objective (oil). All images were captured in bright-field mode and analyzed using ImageJ.

### Turbidity Measurement

The turbidity titrations were used to determine the critical salt concentration (CSC) of coacervates formed by negatively charged molecules (UTP, ATP, D_100_, and polyA) with either pLL or R_10_, as well as UTP with R_100_. Turbidity was measured on a microplate reader (Tecan Infinite M1000 PRO). Briefly, the turbidity of a coacervate solution with a total starting volume of 100 μL (*C*_NaCl_ = 0 M) was monitored as a function of the concentration of NaCl at a wavelength of 600 nm and a temperature of 26 ± 1 °C in 96-well plates (Greiner Bio-one, clear flat-bottom wells) by titration with NaCl (1.5 and 3.0 M) in 1, 2, 5, or 10-μL steps (manually). Samples were incubated for 5 min at the test temperature. After each injection step, the samples were mixed by shaking for 5 s before every readout. The critical point was calculated by extrapolating the first-order derivative at the inflection point to zero turbidity. Note that this critical salt concentration does not take into account ions from other sources than the added NaCl, and the actual critical ionic strength may be slightly higher. The CSC data is summarized in Table S1.

### Surface Charge of Coacervates

Zeta potential measurements were performed using a Malvern DLS-Zetasizer. The positively and negatively charged molecules used to form the coacervates were diluted 2 to 10 times. After coacervates formation, 800 μL of the sample was injected into a disposable folded capillary cell (DTS1070) and measured at 25 °C. Three measurements were taken, each consisting of 100 runs.

### Analysis of the Number of Droplets in the Self-Organized Structures and the Contact Angles

For the automatic image analysis, raw fluorescence confocal microscopy images and videos were processed and analyzed with MATLAB 2021 image Processing Toolbox. Bright and dark objects were isolated and masks for chains of connected droplets were constructed, after which masks smaller than 25 pixels and individual droplets touching the border were removed. For each chain, the sum of droplets was calculated by adding the number of bright and dark droplets inside the mask together. The length of chain in pixels was calculated by finding the nearest neighbors of each bright droplet. In short, the bright droplet was dilated and the Euclidian distance to overlapping dark droplets was recorded using the centroids resulting from a fit of a circular bounding box around each droplet. When no neighbor was found, the droplet was further dilated until a dark droplet was found. The bond angle ( π−θ ) was calculated when coacervate droplets had two or more neighbors, constructing two vectors from the centroid of one neighbor to the central droplet, and from the central droplet to the centroid the other neighbor and calculating the angle using the dot product.

Contact angle analysis was based on Guzowski et al.^17^ Contact angles were calculated using the mask previously determined and fitting a minimum bounding circle to the individual droplets. Bounding circles that consisted of <50% masked area were removed. A third circle was fitted to three specific points: the two intersection points of the bounding circles of neighboring droplets, and the contact point between the centers of the two droplets. Angles between the bounding circles at the intersection points were used to calculate the contact angles.

For the analysis of the number of droplets per chain and the chain length of the chain-like structures induced by mCherry, FtsZ, and PEG_1k_-*b*-pGlu_100_, the *Kappa* plugin in ImageJ was used to measure curvature as a proxy for chain length. Droplet counts were performed manually. All corresponding data are summarized in Table S2. Additionally, contact angles of the chain-like structures formed upon the addition of mCherry, FtsZ, and PEG_1k_-*b*-pGlu_100_ (in Figure 6) into the multiphase coacervates were measured manually using the *Angle* plugin in ImageJ. These data are also summarized in Table S2.

**Figure 6:**
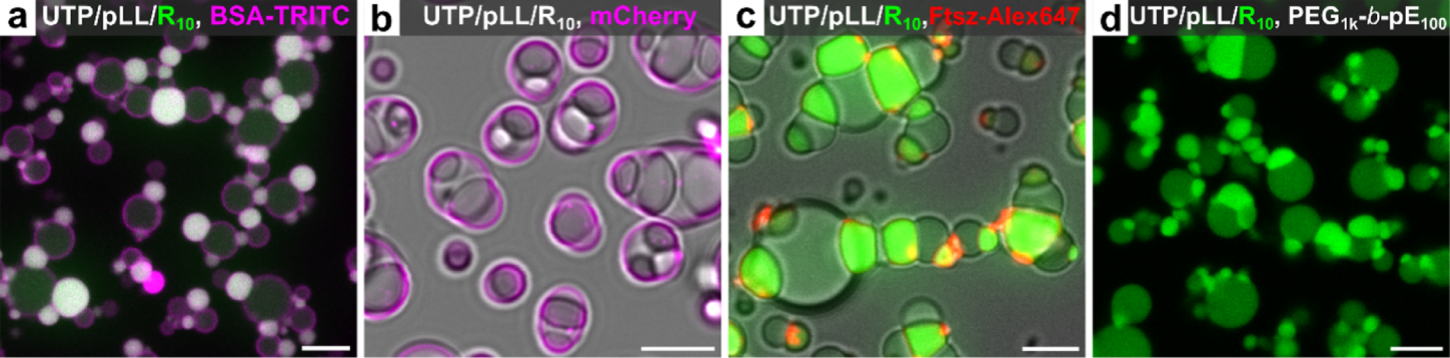
Regulation of multiphase coacervate partial wetting and self-organization by various interfacial macromolecules (mostly proteins). Composite confocal images of (a-c) negatively charged proteins and (d) a negatively charged block copolymer added to UTP/pLL/R_10_ multiphase coacervates: (a) BSA-TRITC at 2.5 μM, *t* = 35 min; (b) mCherry at 0.36 μM, *t* = 30 min; (c) FtsZ at 2.0 μM, *t* = 50 min; and (d) PEG_1k_-*b*-pGlu_100_ (PEG_1k_-*b*-pE_100_) at 3.0 μM, *t* = 37 min. Fluorescence is shown from FAM-R_10_ (green), BSA-TRITC and mCherry (magenta), and AF647-labeled FtsZ (red). All scale bars represent 5 μm.

## Results and Discussion

### αSyn Induces Multiphase Coacervate Dewetting

We form multiphase model coacervates with droplet-in-droplet architecture by mixing UTP/R_10_ complex coacervates (core phase) with UTP/pLL coacervates (shell phase), shown in Figure 1a-c. Upon introducing αSyn (10.7 μM) to these multiphase coacervates, we observe a gradual localization (over 5 min) of αSyn at the interface of the shell phase, then to the core–shell interface, and finally also partitioning into the UTP/R_10_ core phase (Figures 1d and S1a,b). Time-dependent αSyn accumulation at each location is shown in Figure S1c. This process coincides with the UTP/R_10_ core phase moving out of the UTP/pLL shell phase, eventually forming a snowman-shaped structure, leading to a 14% (white arrows indicated) to 22% (magenta arrows indicated) reduction in the diameter of the outer phase droplets (Figures 1d, S1a,b and Movie S1).

αSyn thus induces a wetting transition in the multiphase coacervates from complete wetting, with the core phase completely surrounded by shell phase, to partial wetting, with the core attached to its formed shell. The αSyn-induced wetting transition is primarily due to its affinity for the coacervate interface^13^ and the attractive interaction between the negatively charged tail of αSyn and the positively charged multiphase coacervates of UTP/pLL/R_10_ (ζ-potential = +25.4 ± 0.6 mV). This is supported by control experiments with a truncated variant of αSyn that lacks the negatively charged C-tail (αSyn_1–108_). When αSyn_1–108_ was introduced into UTP/pLL/R_10_ multiphase coacervates, the multiphase structure remained intact without forming partially wetted structures (Figure S2).

In single-phase coacervate systems, αSyn has also been observed to bind to the positively charged surfaces of both UTP/pLL (Figure S1d) and UTP/R_10_ (Figure S1e) coacervates, respectively. We find that this binding alters the interfacial tension (determined through contact angle analysis of β_UTP/pLL_ and β_UTP/R10_) ratios between the coacervate and dilute phases: γ_1d_/γ_12_ remains almost stable over time, while γ_2d_/γ_12_ gradually increases (Figure 1e). We attribute this to a stronger decrease of γ_12_ than γ_2d_ over time, which aligns with the data shown in Figure S1c, where the intensity of AF647-labeled αSyn at the core/shell interface increases rapidly after 100 s, while the intensity of AF647-labeled αSyn at UTP/pLL (droplet 2) interface has reached a plateau around that time. This shift coincides with the observed wetting transition from a nested multiphase structure to a partially wetting morphology.

### αSyn Promotes Self-Organization of Coacervates into Higher-Ordered Polymer-Like Structures

The spontaneous bottom-up assembly of higher-order cytomimetic systems remains a challenge in synthetic biology. Recent research by the Qiao and Mann groups achieved bottom-up self-organization of ordered protocell networks by mixing cationic surfactant didodecyldimethylammonium bromide, cationic polymer poly­(diallyldimethylammonium chloride), and a photoactive anionic aspartic-acid-appended azobenzene derivative. This mixture produces a binary population of immiscible coacervates that can spontaneously self-organize into linear and branched chains at room temperature.^14^ Additionally, in the studies by Gibson and Liu, demixed coacervates were also observed attaching to each other to form a chain-like structure.^7,10^ They described this as a new demixed liquid phase rather than a higher-order structure. However, the chain-like structure of demixed DNA droplets in Liu’s paper phase transitions to a multiphase structure over time.^10^

Here, upon adding αSyn to the UTP/pLL/R_10_ multiphase model coacervates (αSyn = 10.7 μM), the exposed UTP/R_10_ core droplets can attach to neighboring UTP/pLL shell coacervates, linking shell and core coacervates together. Over time, this results in the formation of chain-like or network-like higher-order structures with an alternating sequence of UTP/pLL and UTP/R_10_ droplets, which are reminiscent of polymers but made of coacervates (Figures 2a, S3 and Movie S2). These interconnected coacervate networks emerge within 20 min of αSyn addition and remain locally dynamic, akin to a chain of connected (colloidal) beads or a polymer chain.^18^

Figure 2b shows a stepwise view of the self-organization process. αSyn induces UTP/R_10_ droplets to either wet the inner surface of the UTP/pLL phase or to be released and attach externally to the UTP/pLL phase (120 s). The wetted UTP/R_10_ core droplets (indicated by magenta arrows and numbers) then link with UTP/pLL shell droplets (indicated by cyan arrows and white numbers), a process that repeats for other droplets. Simultaneously, droplets of the same type can fuse with each other, as evidenced by the coalescence of UTP/pLL droplets (indicated by two facing cyan arrows, at t_0_+290 s) and UTP/R_10_ droplets (indicated by two facing magenta arrows, at t_0_+290 s). Ultimately, the partially wetted coacervates self-organize into chain- and network-like complex structures. Time-dependent plots of self-organization structure in Figure 2c show that the mean number of droplets in each chain increases over time (ca. 1200 s). The formation of chains and network-like structures is strongly dependent on the stoichiometry of the two coacervate types. In samples prepared with unequal amounts of UTP/pLL and UTP/R_10_ (UTP/pLL: UTP/R_10_ = 1:4, 1:2, 2:1, and 4:1), only very short chains or partially wetted structures were observed, even after 1 h (Figure S4).

### The Concentration of αSyn Impacts the Organization of Multiphase Coacervates

Based on the results in Figure S5, it is evident that varying concentrations of αSyn introduced into UTP/pLL/R_10_ multiphase coacervates influenced the partial wetting between core and shell coacervates. We hypothesize that the partial wetting structure in the early stage of multiphase coacervate formation (Figures S5 and 6) may influence the subsequent organization into higher-order chain-like structures. To explore this, we examined the effect of different αSyn concentrations on the formation of chain-like structures (Figure 3a). At a low αSyn concentration of 2.7 μM (Figures 3a1 and S7), the multiphase coacervates formed shorter chain structures. The self-organization process (Figure S7) at this concentration is similar to that observed at 10.7 μM (Figure 2), where the partially wetted UTP/R_10_ core droplets self-organize with UTP/pLL shell droplets, and similar droplets can coalesce.

However, due to the lower αSyn concentration, the interfacial tension between UTP/R_10_ core and UTP/pLL shell droplets did not change significantly, allowing for the simultaneous presence of partially wetted structures (at t_0_+310 s), multiphase coacervates (e.g., the engulfing process t_0_+460 s to t_0_+470 s), and chain-like morphologies (at t_0_+2650 s) (Figure S7). This coexistence resulted in shorter chain structures. As the αSyn concentration increased, we observed the formation of longer chains, more branched chains (Figure 3a2–4), and even network structures (Figure 3a5, αSyn = 21 μM). However, with a further increase in αSyn concentration (αSyn = 40 μM), the previously observed chain- and network-like structures contracted into multiphase coacervates, forming a αSyn-rich phase with αSyn localized at the interface of the shell phase of the UTP/pLL droplets (Figures 3a6 and S8b).

To analyze the relationship between αSyn concentration and the characteristics of the self-organized coacervate chains (coacervate polymer), we plotted the average number of droplets per chain, which increases over time (Figure 3b). The average number of droplets per chain increased the fastest at an αSyn concentration of 21 μM. Figure 3c further shows that both the maximum number of droplets in the self-organized structures and their size (chain length) increase with increasing αSyn concentration, reaching a maximum of around 700 connected droplets (see Figure S9), with a total length of 3 mm at a concentration of 21 μM, and then suddenly drop at 40 μM αSyn, where we observed a large multiphase droplets with multiple cores (Figure 3a6). This trend correlates with a gradual change in interfacial tensions (Figure 3d).

The average interfacial tension ratios (γ_1d_/γ_12_ and γ_2d_/γ_12_) in self-organized structures decrease with increasing αSyn concentration (Figure 3d). This trend indicates that the morphologies transition from partial wetting to nearly nonwetting, attributed to a faster decrease in interfacial tension in the core phase (UTP/R_10_, droplet 1) than in the shell phase (UTP/pLL, droplet 2). We also analyzed the angle θ between the vectors connecting neighboring coacervates (indicated in Figure 3a) in the coacervate polymers (Figure S10), which represents the deviation from a perfectly straight chain, and found that the angle θ was significantly smaller than 90 ° $\left(\mathrm{\theta ̃}=60^{o}\right)$ , thus favoring the formation of extended chains of connected coacervates. The observed chain-like structures are reminiscent of polymer structures or colloidal chains, which also exhibit angular restrictions, and the angle θ is analogous to the angle between neighboring bonds, or π–θ to the bond angle.^18,19^ We will discuss the origin of the observed centroid angle distribution in the next section. Finally, Figure S10 showed that the αSyn concentration had no significant effect on the bond angles.

We verified that the formation of chain-like structures was not the result of a kinetic trapping by preparing samples, in which αSyn was added at different points. Chain-like structures were consistently observed regardless of when αSyn was introduced: after the formation of UTP/pLL/R_10_ multiphase coacervates (Figure 3a2), premixed with UTP/R_10_ coacervates and prior to the addition of UTP/pLL coacervates (Figure S11a-c), or separately added to UTP/pLL and UTP/R_10_ coacervates before they were mixed (Figure S11d-f).

### Angle Dynamics of the Multiphase Coacervates

To better understand the striking transformation of multiphase coacervates to chains and network-like structures of connected immiscible coacervates upon the addition of a surface-localizing protein, we developed a minimal theoretical model consisting of four components: a solvent, two droplet-forming components (A and B, reminiscent of UTP/pLL and UTP/R_10_, respectively), and an interfacial species α (representing an interfacial protein, such as αSyn) that is miscible with all other components and is enriched at the interface between the different phases. We describe the free energy *F* of the system:

$$F=\int dx\frac{k_{B}T}{\nu }\left[f\left(\phi_{A},\phi_{B},\phi_{\alpha }\right)+\frac{\kappa_{A}}{2}{\left(\nabla \phi_{A}\right)}^{2}+\frac{\kappa_{B}}{2}{\left(\nabla \phi_{B}\right)}^{2}+\frac{\kappa_{\alpha }}{2}{\left(\nabla \phi_{\alpha }\right)}^{2}+\sigma_{A}\nabla \phi_{A}\nabla \phi_{\alpha }+\sigma_{B}\nabla \phi_{B}\nabla \phi_{\alpha }\right]$$ 1

where $\phi_{i}\;\left(i=A,B,\alpha \right)$ are the volume fractions of the droplet-forming components and the interfacial species, $k_{B}T$ is the thermal energy, and ν the solvent molecular volume. For the mixing free energy density f , we use a Flory–Huggins model as detailed in the Supporting Information (section 2: Supplementary theory). The constants $\kappa_{A},\kappa_{B},\kappa_{\alpha }$ account for an energetic penalty for inhomogeneities in volume fractions and are related to the surface tensions.^20^ The last two terms in eq 1), with the two dimensionless constants σ_A_ and σ_B_, only contribute dominantly at the coacervate interfaces and promote the enrichment of α at the interfaces. The dynamics of the phase fields, follow Cahn–Hilliard equations, $\frac{\partial \phi_{i}}{\partial t}=\nabla \left[\Lambda_{i}\nabla \mu_{i}\right],i=A,B,\alpha$ , where the chemical potentials $\mu_{i}=\nu n_{i}\frac{\delta F}{\delta \phi_{i}}$ are obtained from the free energy (eq 1), with $n_{i}$ the ratio of the molecular volume of component i and the solvent. The mobilities $\Lambda_{i}$ are modeled as $\Lambda_{i}=\Lambda^{\left(0\right)}\phi_{i}\left(1-\phi_{A}-\phi_{B}-\phi_{\alpha }\right)$ , with $\Lambda^{\left(0\right)}=const$ . We solve the Cahn–Hilliard equations numerically for a two-dimensional system. Details about the numerical simulations are summarized in the Supporting Information (section 2).

This minimal model can reproduce key experimental observations when we vary the concentration of interfacial species α . In the absence of α , we find nested coacervates. As the volume fraction of α in the outer, dilute phase is increased from $\phi_{\alpha }=0.001$ to $\phi_{\alpha }=0.01$ , the coacervates show a transition to partial wetting (Figure 4a), coinciding with a pronounced enrichment of α at the coacervate interfaces and in the core (B-rich) phase (cf., Figures 1d and S1b, c).

In coacervate chain formation, the transition to partial wetting corresponds to the formation of coacervate dimers, which can connect with neighboring dimers to form extended polymer-like chains in alternate order. To understand why predominantly extended chains are formed with obtuse angles between vectors connecting neighboring coacervates (θ < 90 °, cf., Figure S10), we investigate the next larger unit: coacervate trimers (Figure 4b). While experimental access to the dynamics of neighboring coacervates is limited, as they rapidly assemble into networks, numerical simulations allow us to analyze their behavior immediately after the first contact of two coacervates. We consider an A-rich coacervate between two B-rich coacervates, where the size and arrangement of the coacervates is symmetric (Figure 4b). Interestingly, the coacervate arrangement straightens. Minimizing the surface energy drives the A-rich coacervate to slide between the B-rich coacervates. In contrast, the outer B-rich coacervates shift only marginally during the straightening process due to their significantly lower mobility, which is about nine times smaller than that of the A-rich phase in our simulation.

To establish a link to polymer-like structures, we discuss the dynamics of the system in more detail. We denote by $2D_{0}$ the distance between the outer coacervates when all three coacervates are aligned in a straight line. If the center of the middle coacervate is displaced, the surface increases and the surface tension generates a restoring force. As the center coacervate moves to straighten the arrangement the distance between the centers of adjacent coacervates D decreases toward $D=D_{0}$ . To linear approximation, the change of the contact area between the coacervates and thus the change in surface energy scales with $∼\left(\gamma_{12}-\gamma_{1d}-\gamma_{2d}\right)\left(D-D_{0}\right)$ . Here, $D-D_{0}$ is linked to the angle θ formed by the neighboring droplet dimers via $D\;\cos\left(\theta /2\right)=D_{0}$ . We expand the change in surface energy for small angles θ upon displacement of the center coacervate and obtain

$$\Delta E∼\left(\gamma_{12}-\gamma_{1d}-\gamma_{2d}\right)D_{0}\left[\frac{1}{2}{\left(\frac{\theta }{2}\right)}^{2}+\frac{5}{24}{\left(\frac{\theta }{2}\right)}^{4}\right]$$ 2

For small deviations to a straight alignment, the dynamics of the angle follows $\frac{\partial \theta }{\partial t}∼\frac{\partial \Delta E}{\partial \theta }$ . The resulting time evolution of the angle can be determined analytically:

3 $$\theta \left(t\right)=\theta_{0}\frac{2}{\sqrt{\left(4+\frac{5}{6}\theta_{0}^{2}\right)\exp\left[\frac{t}{2\tau_{\mathrm{rel}}}\right]-\frac{5}{6}\theta_{0}^{2}}}$$

with a characteristic relaxation time $\tau_{\mathrm{rel}}$ that agrees with the dynamics of the simulation (Figure 4b).

Now we can make an analogy to an extensible semiflexible polymer. From the simulations we learn that the relaxation is driven by two processes (i) bending relaxation and (ii) shortening of the inter coacervate distance. We consider a coacervate polymer that exhibits a bending stiffness κ , as it is known for wormlike-chains,^21^ and additionally experiences a restoring potential around a segment rest length $b_{0}$ , similar to a Rouse model.^22^ The corresponding energy of a single polymer segment is ${\Delta E}_{\mathrm{seg}}=\frac{\kappa }{2}\epsilon^{2}+\frac{k}{2}{\left(b-b_{0}\right)}^{2}$ , with ϵ the angle between neighboring segments, k a spring constant and b the segment length. For the deflection from the energy minimum at ϵ=0 and $b=b_{0}$ , b and ϵ are connected via $b_{0}=b\cos\epsilon$ We expand again for small angles ϵ and obtain

4 $${\Delta E}_{\mathrm{seg}}=\frac{\kappa }{2}\epsilon^{2}+\frac{3{\mathrm{kb}}_{0}^{2}}{5}\frac{5}{24}\epsilon^{4}$$

which has the same formal structure as the energy of the coacervate trimers, eq 2. If we now make the analogy θ/2→ϵ and $D_{0}\to b_{0}$ , we can model a string of multiphase coacervates as an elastic polymer with bending stiffness $\kappa ∼\left(\gamma_{12}-\gamma_{1d}-\gamma_{2d}\right)$ and Rouse-like intermonomer distance fluctuations characterized by a harmonic spring constant $k∼\frac{\gamma_{12}-\gamma_{1d}-\gamma_{2d}}{D_{0}^{2}}$ , where we neglect constant prefactors. Notably, the bending stiffness and spring constant are not independent but are both set by the surface tension of the coacervates. From these observations, we anticipate that for larger structures, the alternating arrangement of coacervates promotes chain straightening. This mechanism maintains separation between coacervates of the same type, reducing the likelihood of fusion and enabling the formation of extended chains, similar to observations in other multiphase coacervate systems.^14^

### Controlling Self-Organized Multiphase Coacervates by Modulating Interfacial Adsorption

αSyn alters the surface tension of coacervates and stabilizes the droplets.^13^ We hypothesized that removing αSyn from the droplet interface could revert the chain-like structure back to a multiphase coacervate or a homogeneous droplet. To examine whether such dynamic control is feasible, we introduced negatively charged small molecules like ATP and longer polymers such as poly-l-aspartic acid (D_100_) and polyA into the self-organized coacervates. We also tested positively charged molecules, including positively charged surfactant hexadecyltrimethylammonium bromide (CTAB), block copolymers PEG_5k_-*b*-pLL_100_, and peptide poly-l-arginine R_100_, all capable of interacting with αSyn or participating in the coacervates formation.

Figure 5 illustrates the effect of these coacervate surface modifiers: adding αSyn (5.4 μM) to UTP/pLL/R_10_ multiphase coacervates formed chain-like structures within 20 min (Figure 2a). However, further addition of negatively charged molecules (ATP (0.5 mM), D_100_ (2.5 mM), and polyA (0.3 mg/mL)) caused these chain-like structures to revert to multiphase coacervates (as indicated by the dotted circles and white arrows) (Figures 5b,c, S12a, and S13a). Since all these negatively charged molecules can form more stable coacervates with both cationic peptides pLL and R_10_ compared to UTP (Table S1), their addition to the self-organized structures leads to their partitioning into the UTP/pLL and UTP/R_10_ coacervates within the chains. This likely alters the composition of those droplets, making their surfaces less positively charged.^23^ As a result, the interactions between αSyn and coacervate surfaces decrease, causing αSyn to detach from the coacervate interface (scheme in Figure 5a). This alters the coacervate interfacial tension, prompting the chain-like structures to revert to a multiphase coacervate state. For example, this phenomenon is clearly observed in an experiment where D_100_ is added after the multiphase coacervates have self-organized into chain-like structures (Figure 5c and Movie S3). Only a few droplets remain associated with αSyn, while most appear depleted of αSyn, and αSyn in the dilute phase exhibits high fluorescence intensity (Figure S13b, αSyn channel). Meanwhile, introducing negatively charged ATP and D_100_ leads to multiphase coacervate phase inversion, causing UTP/R_10_ to shift from the inner phase to the shell phase (Figure 5b,c), as both ATP/pLL and D_100_/pLL have higher critical salt concentrations than UTP/R_10_ (Table S1).

Interestingly, introducing positively charged surfactant or polymers (CTAB (0.5 mM), PEG_5k_-*b*-pLL_100_ (2.5 mM), and R_100_ (2.5 mM)) into the chain-like structures (Figures 5d,e and S12b, S13c,d) leads to varied outcomes. Both CTAB and PEG_5k_-*b*-pLL_100_ reverse the chain-like structure back into multiphase coacervates (Figures 5d and S12b, S13c), similar to the effect of adding negatively charged molecules. By contrast, the addition of R_100_ transforms the chain-like structure into homogeneous droplets (Figure 5e, marked by white arrows). The results indicate the following process, as outlined in the scheme (Figure 5a): positively charged surfactant or block copolymer molecules (or micelles) bind to αSyn, forming a complex that removes αSyn from the UTP/pLL and UTP/R_10_ interfaces. This alters the coacervate interfacial tension, leading to the breakdown of the chain-like structure and a return to multiphase coacervates, as seen when CTAB and block copolymers like PEG_5k_-*b*-pLL_100_ are added (Figures 5d and S12b, S13c). The cationic peptide R_100_ has a stronger interaction with UTP than with R_10_ (Table S1), and it strongly partitions into the UTP/pLL and UTP/R_10_ coacervates, thereby liberating pLL and R_10_. This ultimately causes UTP/pLL and UTP/R_10_ coacervates to merge within the chain-like structure, resulting in single-phase/homogeneous coacervates (Figures 5e and S13d). This process is reminiscent of the docking and fusion observed in P-bodies and stress granules.^5^ We note that the average area of the merged homogeneous coacervate is about 32% larger than the area of the original core, but smaller than the combined areas of the original core and shell, because the stronger interactions of R_100_ with UTP leads to denser coacervates. It is also possible that the shell phase of UTP/pLL partly disintegrates due to the strong interaction between R_100_ and UTP.

These results indicated that αSyn adsorption at the self-organized coacervate interface is dynamic and can be disrupted by the introduction of other negatively or positively charged molecules. This finding provides a way to modulate the structural organization of multiphase coacervates.

### Various Interfacial Proteins Can Regulate Multiphase Coacervate Partial Wetting and Self-Organization

To investigate whether proteins other than αSyn can also localize at the interface of multiphase coacervates, inducing a wetting transformation and self-organization into higher-order structures, several disordered proteins (BSA, mCherry, and FtsZ) with negatively charged domains were selected for incorporation into the UTP/pLL/R_10_ multiphase coacervates. Additionally, a negatively charged block copolymer PEG_1k_-*b*-pGlu_100_ was also examined (Figures 6 and S14–S17).

TRITC-labeled BSA (BSA-TRITC) behaves similarly to αSyn, attaching to the UTP/pLL shell interface and partitioning into UTP/R_10_ core coacervates. This induces the partial wetting and release of core droplets (UTP/R_10_), which then self-organize with the shell phase into alternately connected chain-like structures (Figures 6a and S14a–S16a). On the other hand, mCherry induces partial wetting without forming extensive chain-like structures, typically linking only three or four droplets (Figures 6b and S14b, S16b). Interestingly, for FtsZ we observed patchy interfacial localization, possibly because of FtsZ bundling. Nevertheless, it still caused core droplet (UTP/R_10_) escape and the formation of chain-like structures (Figures 6c and S14c). Finally, the nonfluorescent block copolymer PEG_1k_-*b*-pGlu_100_ also induces UTP/pLL/R_10_ partial wetting and the formation of chain-like structures (Figure 6d). We hypothesize that the common feature between these interfacial proteins and PEG_1k_-*b*-pGlu_100_ is their amphiphilicity. They all contain both hydrophobic and hydrophilic regions and carry negative charges, enabling interactions with positively charged molecules or coacervate surfaces.

Quantitative analysis indicates that most interfacial proteins preferentially localize at the interface between the core (UTP/R_10_) and shell (UTP/pLL) droplets (Table S2), where they play a central role in driving the formation of chain-like structures. This interfacial accumulation modulates the interfacial tension between coacervate phases, thereby altering the contact angle and promoting partial wetting and chain-like structure formation. The local concentration at the core/shell and shell/solution interface appears to be critical to determine the chain length: proteins with weaker localization, such as mCherry, formed relatively short chains. In contrast, proteins that do not localize to the interface but only partition into the coacervates, such as eGFP (Figure S17a,b), do not induce partial wetting or support the formation of chain-like assemblies; instead, the multiphase structure is preserved. To assess the importance of interfacial protein localization, we conducted simulations in which the parameters promoting interface accumulation were set to zero $\left(\overset{\sim }{\sigma_{A}\;}=0;\overset{\sim }{\sigma_{B}\;}=0\right)$ . Under these conditions, the limited contact area between the core droplet and the surrounding dilute phase was insufficient to support the formation of distinct dimers or extended coacervate polymers (Figure S17c), in agreement with our experiments.

To utilize interfacial proteins for modifying coacervates in the context of synthetic cells and tissues —particularly for regulating cell–cell interactions and communication—the structural integrity of coacervate/condensate connections must be stable. Accordingly, the stability of the chain-like structures formed by introducing interfacial proteins (αSyn or TRITC-labeled BSA) into UTP/pLL/R_10_ multiphase coacervates at room temperature was assessed. These structures remained intact for over 3 days without reverting to their original multiphase state. We note that in the case of αSyn specifically, the formation of protein aggregates after a lag phase of 10–12 h, did result in an enhanced accumulation of αSyn in the coacervates, but not to a destabilization of the chain-like structures (Figures S18 and S19). BSA-TRITC remained localized at the interface (Figure S18c).

Finally, we examined the salt stability of these chain-like structures. Upon NaCl addition, the UTP/pLL shell phase dissolved (Figure S20), whereas the core (UTP/R_10_) droplets remained stable, and formed a beads-on-a-string morphology. These findings demonstrate that αSyn at the interface plays a critical role in stabilizing the structure and significantly retards coalescence.

## Conclusions

In this paper, we demonstrated that the morphology of UTP/pLL/R_10_ multiphase coacervates can be controlled by addition of proteins that bind to the interface of the coacervates, such as α-synuclein (αSyn). αSyn alters the relative interfacial energies of the coexisting coacervates, leading to a wetting transition from complete to partial wetting of the UTP/R_10_ core droplets. Over time, these partially wetted droplets self-organize into chain- or network-like structures, with the complexity and length of the chains depending on αSyn concentration. The concentration of αSyn affects the average interfacial tension ratios of γ_1d_/γ_12_ and γ_2d_/γ_12_, and ultimately also the length and branching of the coacervate droplet network. The angle dynamics of multiphase coacervates provide insights into their structural transitions. Dimer coacervates undergo a transition to partial wetting, aligning with experiments. In coacervate trimers, surface energy minimization straightens coacervate arrangements a behavior reminiscent of an extensible semiflexible polymer. This mechanism likely extends to larger structures, promoting chain formation while preventing fusion. In addition, the wetting transition and network formation is reversible. By removing αSyn with charged molecules, such as ATP or R_100_, the chain-like structures revert to multiphase or single-phase mixed (homogeneous) coacervates. We observe similar wetting transitions and self-organization with other negatively charged proteins and block copolymer, indicating that these phenomena are general and may play a role in cellular context as well. The stability and reversible formation of these connections provide a foundation for harnessing this mechanism in synthetic cells and tissues to regulate cell–cell interactions and communication.

## Supplementary Material









## Notes

Extended methods, supplementary tables and figures, supplementary theory (PDF)
This video recorded the dynamic process of adding AF647-labeled αSyn into the multiphase UTP/pLL/R_10_ coacervate droplets. The AF647-labeled αSyn molecules interact with the multiphase coacervate interfaces, inducing partial wetting and triggering the release of the inner droplets. In this video, the replay speed is 50 times faster than the recorded experiment (a time stamp is shown at the top left, the total time is 5 min) (Figure 1d)­(AVI)
This video recorded the dynamic self-organization process of UTP/pLL and UTP/R_10_ coacervates following the addition of AF647-labeled αSyn to the multiphase UTP/pLL/R_10_ coacervate droplets. In this video, the replay speed is 100 times faster than the recorded experiment (a time stamp is shown at the top left, the total time is 19 min 10s) (Figure 2)­(AVI)
This video shows the introduction of negatively charged polymer D_100_ into self-organized structures, resulting in the removal of αSyn from the coacervate droplet interfaces and the subsequent reversion of the self-organized structure to a multiphase coacervate state. The video is presented at a playback speed 360 times faster than the actual experiment, with a timestamp displayed in the top left corner, representing a total experimental duration of 60 min (Figure 5c)­(AVI)
