# Supplementary material for: Controlling Multiphase Coacervate Wetting and Self-Organization by Interfacial Proteins
Source: J Am Chem Soc. 2025 Jun 17;147(26):22622–33. doi: 10.1021/jacs.5c03870 (PMC12232295; doi:10.1021/jacs.5c03870)
Supplement: Supplementary file 1 [file ja5c03870_si_001.pdf]

Supplementary information

Controlling Multiphase Coacervate Wetting and Self-Organization by Interfacial Proteins

Tiemei Lu[1,2], Susanne Liese[3], Brent S. Visser[1], Merlijn H. I. van Haren[1], Wojciech P. Lipiński[1], Wilhelm T.S. Huck[1], Christoph A. Weber[3,*] and Evan Spruijt[1,*]

[1] *Institute for Molecules and Materials, Radboud University, Heyendaalseweg 135, 6525 AJ, Nijmegen, The Netherlands*
[2] *Department of Chemistry, University of Oxford, 12 Mansfield Road, OX1 3TA, Oxford, UK*
[3] *Faculty of Mathematics, Natural Science, and Materials Engineering, Institute of Physics, University of Augsburg, Universitätsstr. 1, 86159 Augsburg, Germany*

[*] Correspondence: evan.spruijt@ru.nl; christoph.weber@physik.uni-augsburg.de

Contents

1. **Supplementary Figures** 2
2. **Supplementary theory** 14
3. **Supplementary tables** 16
4. **Supplementary movie captions** 18

1. Supplementary Figures

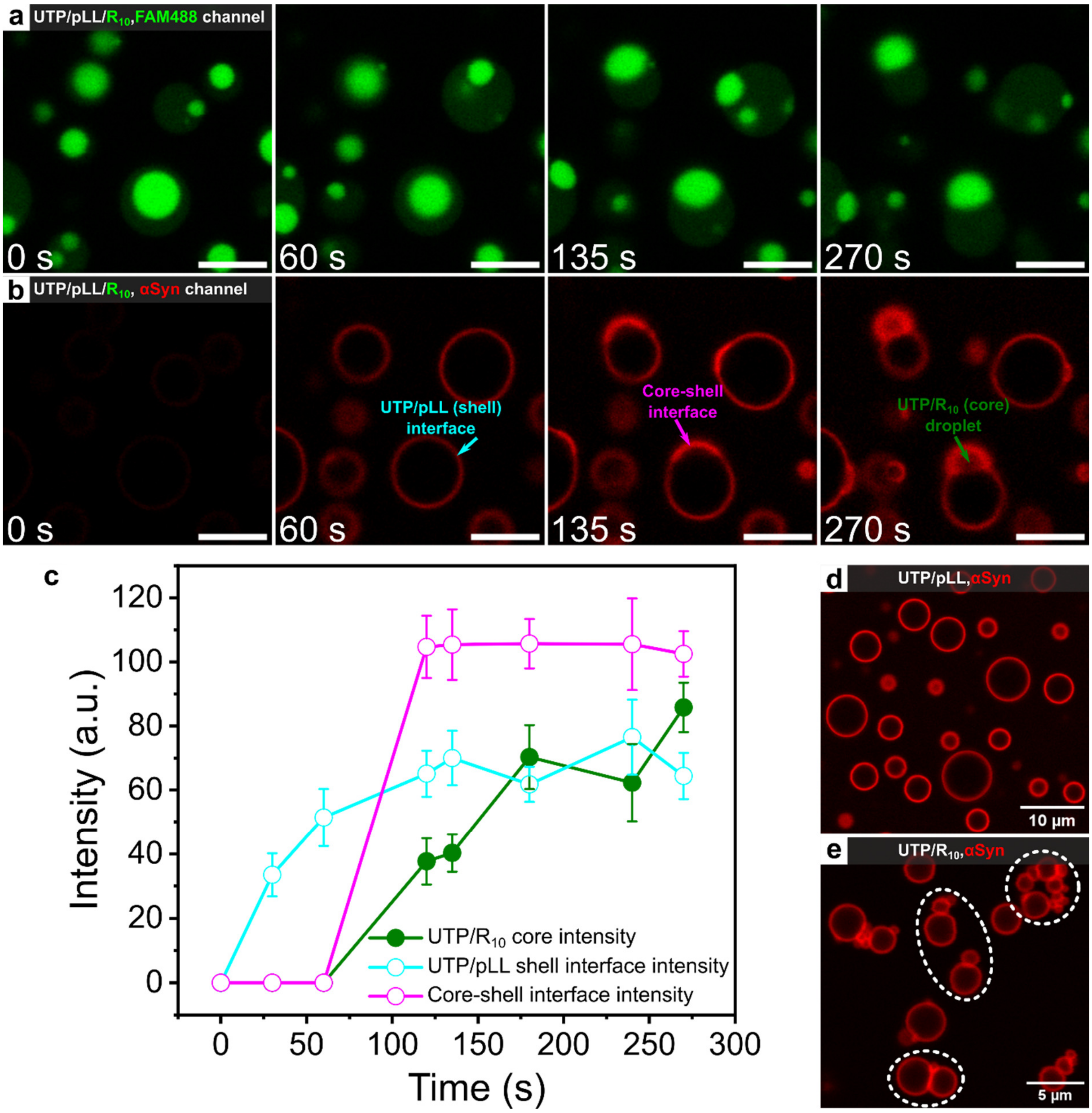

Figure S1: (a,b) Snapshots from confocal fluorescence microscopy illustrating the process of αSyn-induced dewetting in UTP/pLL/R_{10} multiphase coacervates, shown in (a) the FAM 488 channel and (b) AF647 αSyn channel. (c) Average AF647 αSyn intensity over time in the UTP/R_{10} (core) droplets, the UTP/pLL (shell) interface, and the core-shell interface. (d,e) Single complex coacervates of (d) UTP/pLL and (e) UTP/R_{10} after αSyn addition, visualized by confocal fluorescence microscope, with fluorescence from AF647 labelled αSyn. The white dotted line frame in (e) indicates linked UTP/R_{10} coacervates. Scale bars in (a) and (b) are 5 µm.

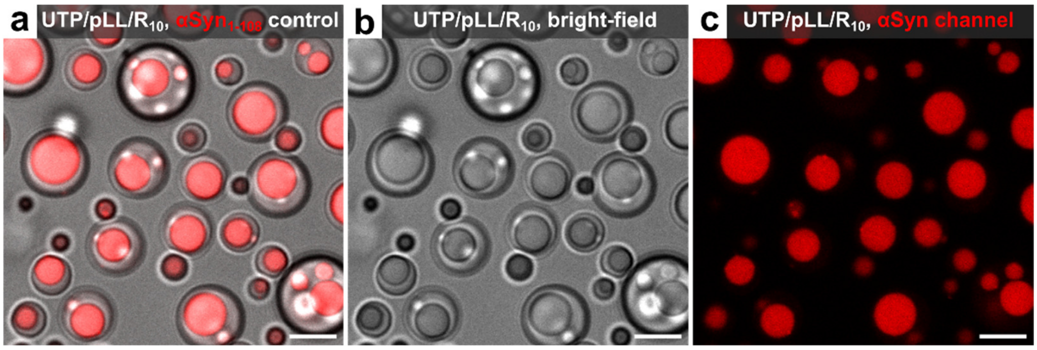

Figure S2: Control experiment with a mutant αSyn$_{1\text{-}108}$ without the C-terminal tail. (a) Composite confocal image of multiphase coacervates UTP/pLL/R_{10} after adding αSyn$_{1\text{-}108}$, with fluorescence from AF647-labelled αSyn$_{1\text{-}108}$ (red), obtained by combining (b) and (c), and showing the separate channels for (b) bright-field and (c) αSyn. αSyn$_{1\text{-}108}$ selectively partitioned into the core phase of UTP/R_{10} coacervates, rather than adhering to the interface of UTP/pLL/R_{10} multiphase coacervates. All scale bars represent 5 µm.

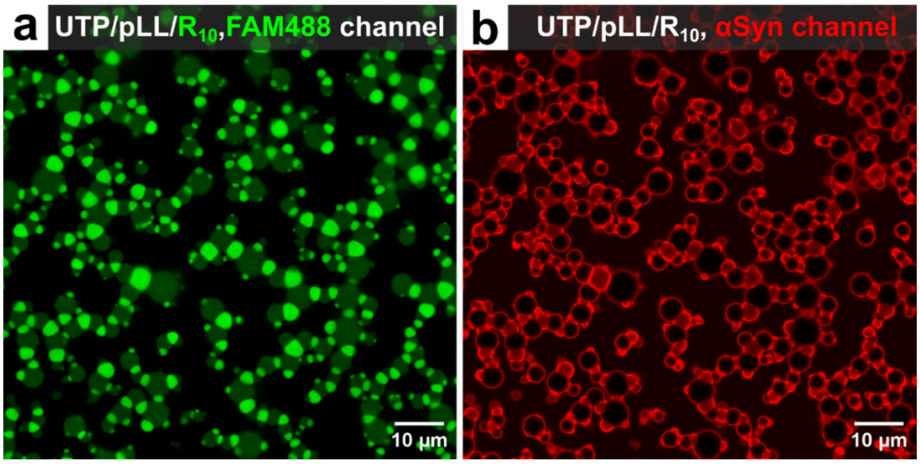

Figure S3. (a,b) Separate channels of UTP/pLL/R_{10} multiphase coacervates after the addition of αSyn, t = 1150s, visualized by fluorescence microscopy. The images show fluorescence from FAM-Ahx-R_{10} (a, green, FAM488 channel) and AF647-labeled αSyn (b, red, αSyn channel).

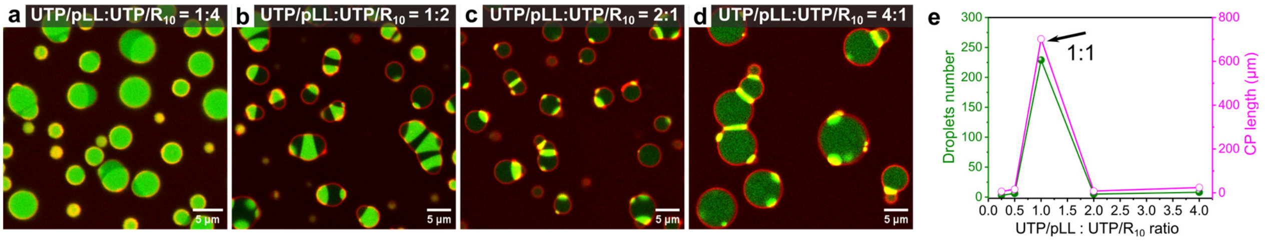

Figure S4. Composite confocal images showing the self-organization of chain-like structures with varying UTP/pLL:UTP/R_{10} ratios in multiphase coacervates: (a) 1:4; (b) 1:2; (c) 2:1; and (d) 4:1, following the addition of αSyn. (e) Quantitative comparison of droplet number per chain and coacervate polymer (CP) length across different UTP/pLL:UTP/R_{10} ratios, indicating optimal chain formation at a 1:1 ratio. Green and red fluorescence correspond to FAM-Ahx-R_{10} and AF647-labeled αSyn, respectively.

αSyn concentration impacts the wetting transition

To obtain a better understanding of the wetting transition of the multiphase coacervates after the addition of αSyn, we investigated the impact of varying concentrations of αSyn (Figure S5a-e). At low αSyn concentration (2.7 μM), αSyn attaches to the shell surface of UTP/pLL coacervates and subsequently partitions into the core UTP/R_{10} coacervates, inducing partial wetting of the core droplets by the shell UTP/pLL droplets (Figure S5a). As the αSyn concentration increases from 2.7 to 21.0 μM (Figure S5b-d), αSyn gradually causes the release of the core UTP/R_{10} droplets from the nested structure. This effect is particularly evident at concentrations of 15.9 μM and 21.0 μM, where a distinct snowman structure is observed, with the two droplets attached to each other (Figure S5c, d). At a further increase in αSyn concentration to 40 μM, following the release of the core droplets, αSyn appears to accumulate at the interface of the core droplets, resulting in the formation of two separate droplets. This result marks a critical transition between non-wetting and mere attachment (Figure S5e).

Given that αSyn exhibited different partitioning in UTP/pLL and UTP/R_{10} coacervates, we investigated whether the order of αSyn addition to multiphase droplets affects their wetting morphologies. To explore this, αSyn was first added to UTP/R_{10} coacervates before introducing UTP/pLL coacervates. In a separate experiment, αSyn was added individually to UTP/pLL and UTP/R_{10} coacervates before mixing them to create multiphase coacervates. When αSyn was first introduced to UTP/R_{10} coacervates and then mixed with UTP/pLL, αSyn localized on the surface and inside UTP/R_{10} coacervates, but they were still able to form partially wetted structures with UTP/pLL (Figure S6a). When αSyn was added separately to both UTP/pLL and UTP/R_{10} coacervates before mixing, partial wetting of UTP/pLL by UTP/R_{10} from the outside was observed (Figure S6b), suggesting that the observed partial wetting morphology is the result of an equilibrium interaction between the different coacervates and αSyn.

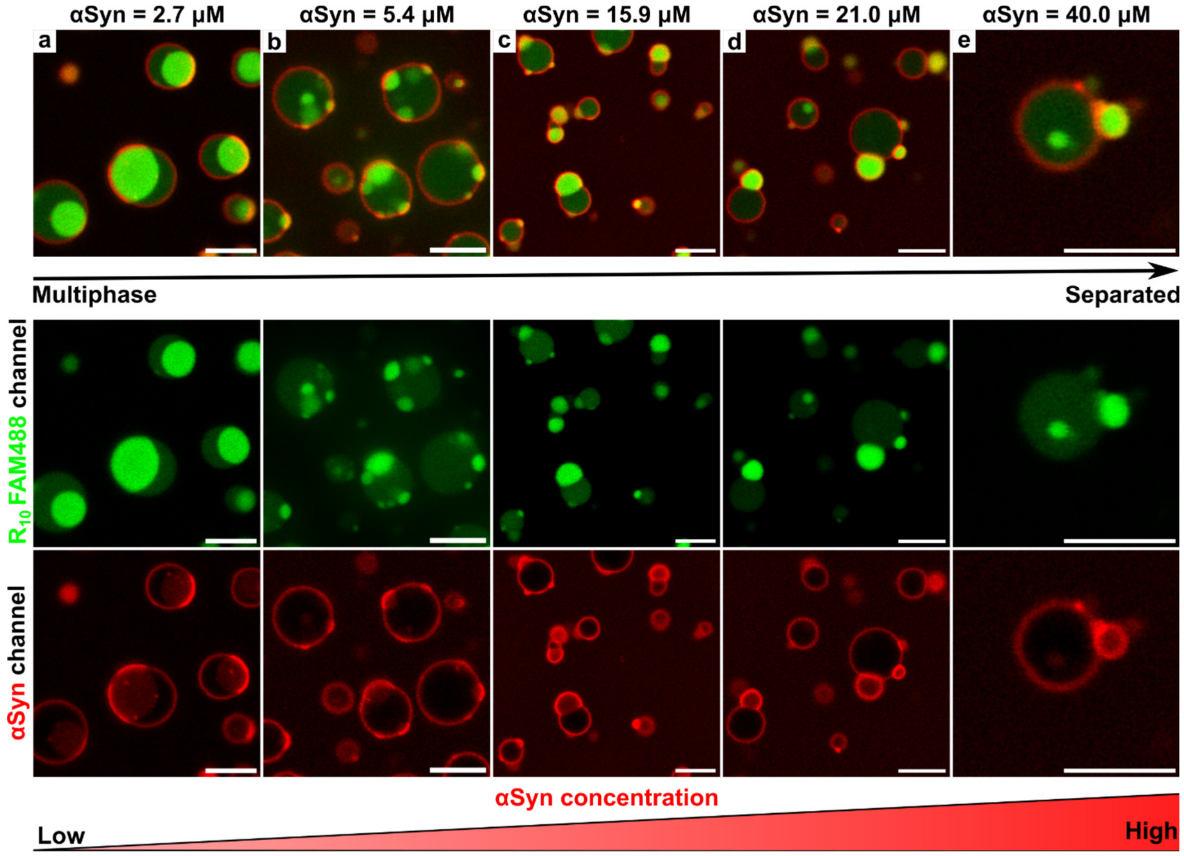

Figure S5. (a-e) Composite confocal images and separate channels of multiphase coacervates UTP/pLL/R_{10} after the addition of various αSyn concentrations, showing fluorescence from AF647-labelled αSyn (red, αSyn channel) and FAM-Ahx-R_{10} (green, FAM488 channel). Images were captured within 2 min of αSyn addition. All scale bars represent 5 μm.

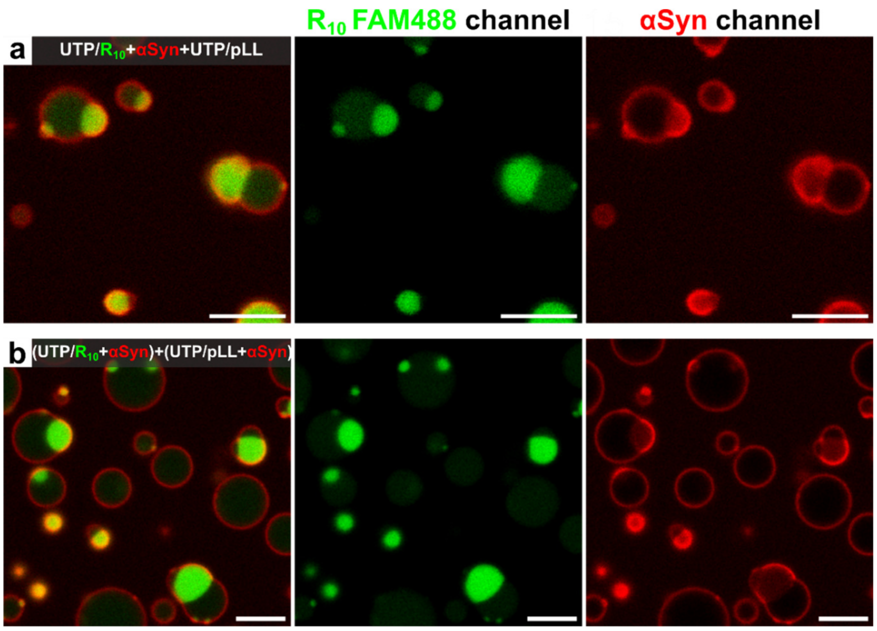

Figure S6. (a,b) Composite confocal images and separate channels showing different αSyn addition sequences into UTP/pLL/R_{10} coacervates. The images show fluorescence from FAM-Ahx-R_{10} (green, FAM488 channel) and AF647-labeled αSyn (red, αSyn channel). All scale bars represent 5 μm.

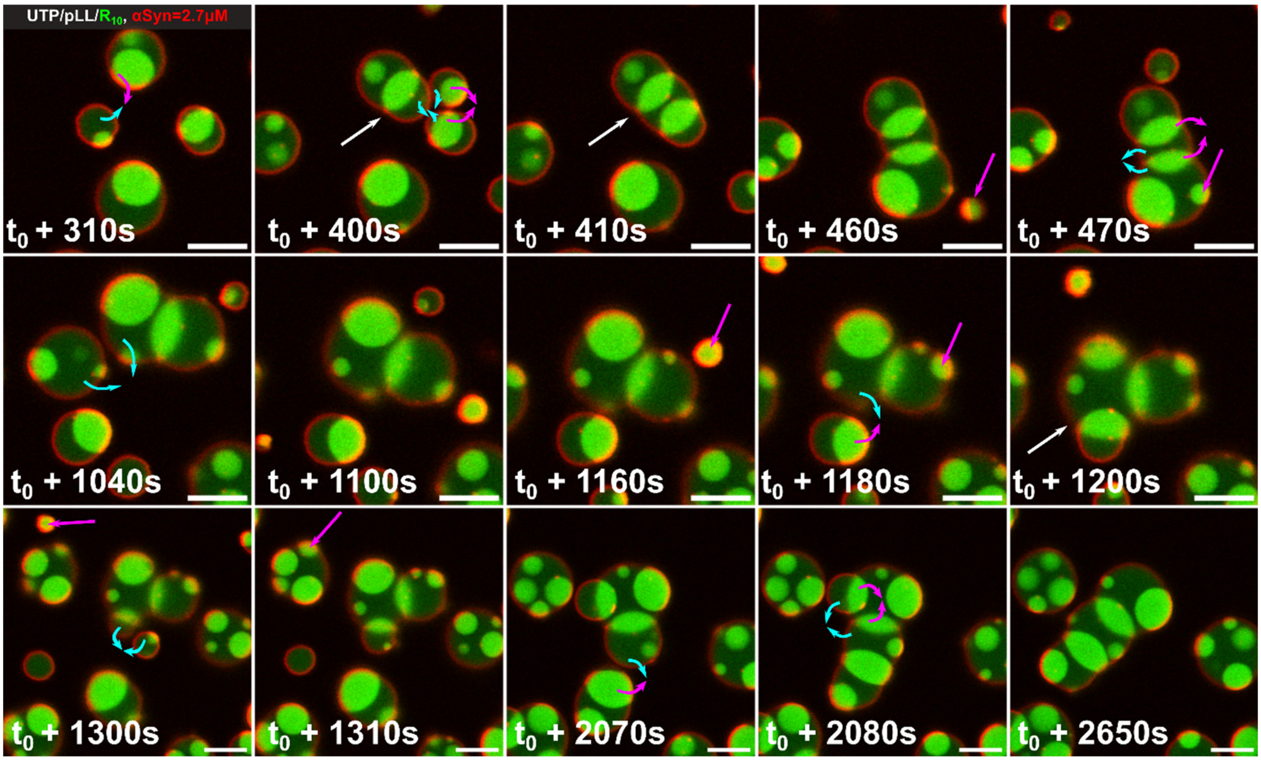

Figure S7. Self-organization of coacervates. Snapshots from confocal fluorescence microscopy, illustrate the αSyn-induced self-organization process of the UTP/pLL/R_{10} multiphase coacervates. Fluorescence from AF647-labeled αSyn (red) and FAM-Ahx-R_{10} (green) is visible. All scale bars represent 5 μm.

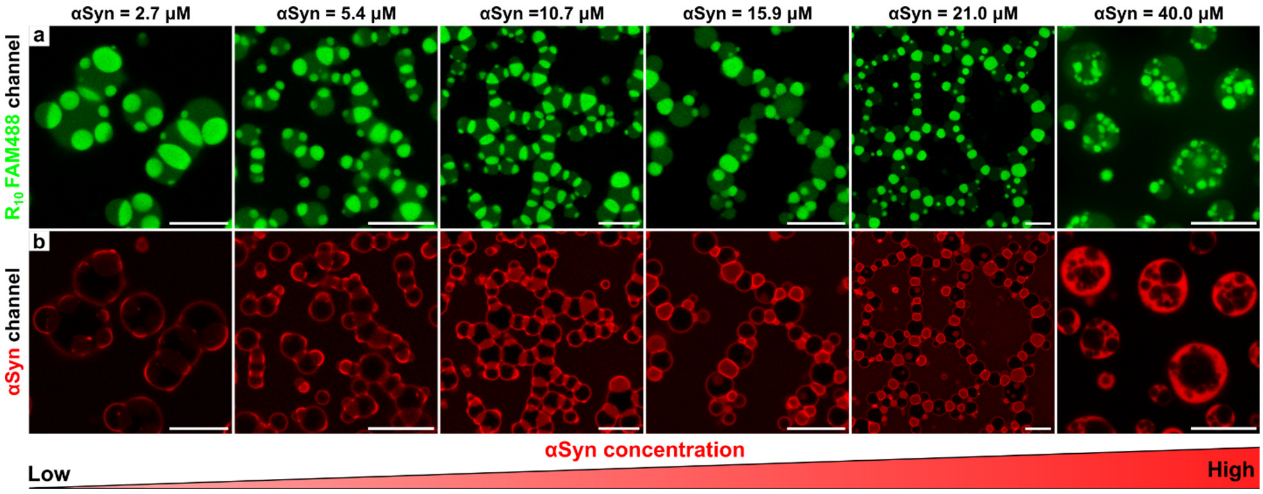

Figure S8. (a,b) Separate channels of UTP/pLL/R_{10} multiphase coacervates, visualized by fluorescence microscopy after the addition of various αSyn concentrations, reveal different self-organization structures. Fluorescence is shown from (a) FAM-Ahx-R_{10} (green, FAM488 channel) and (b) AF647-labeled αSyn (red, αSyn channel). All scale bars represent 10 μm.

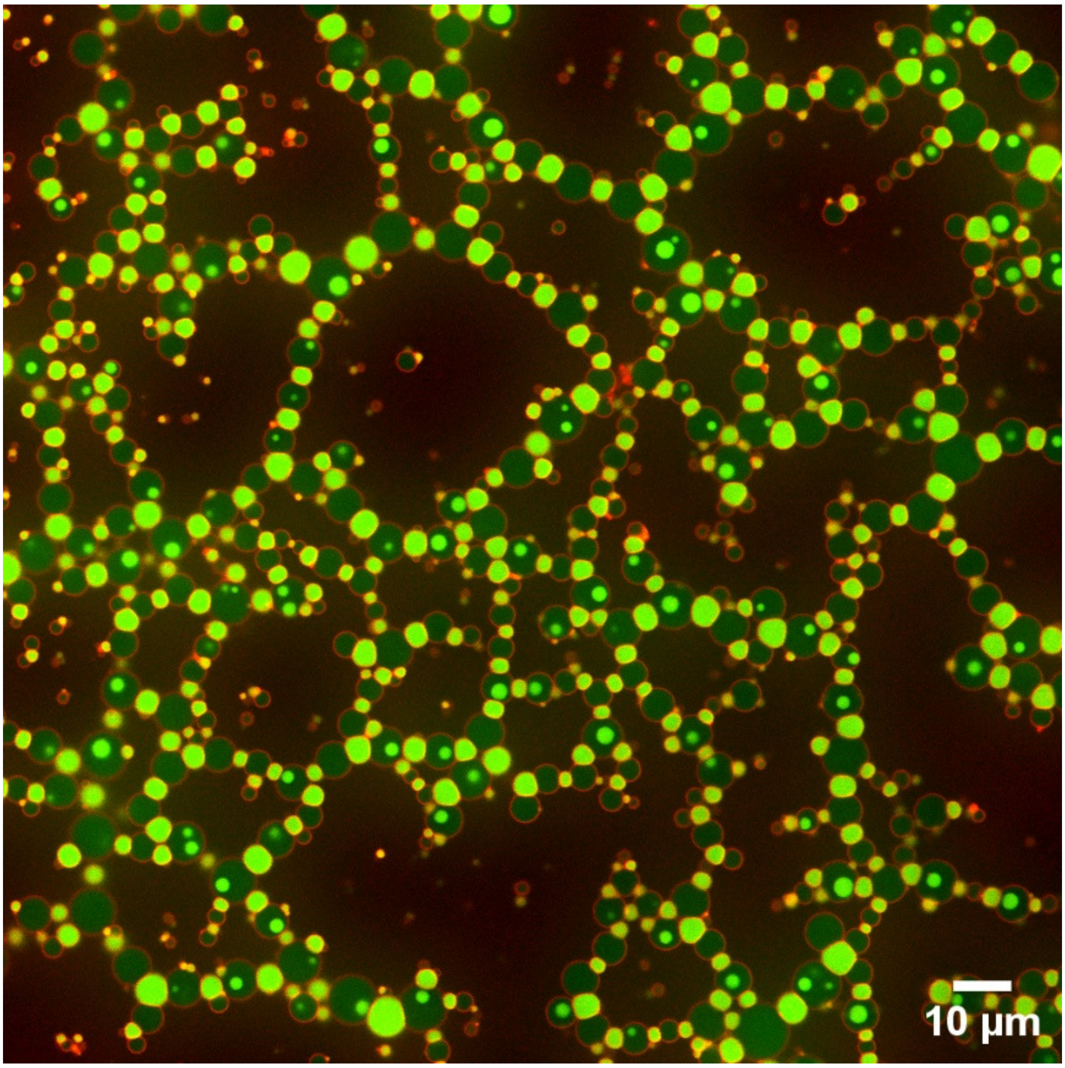

Figure S9. Composite confocal image of a self-organized UTP/pLL/R_{10} multiphase coacervate network, showing 694 interconnected droplets with fluorescence from AF647-labeled αSyn (red) and FAM-Ahx-R10 (green).

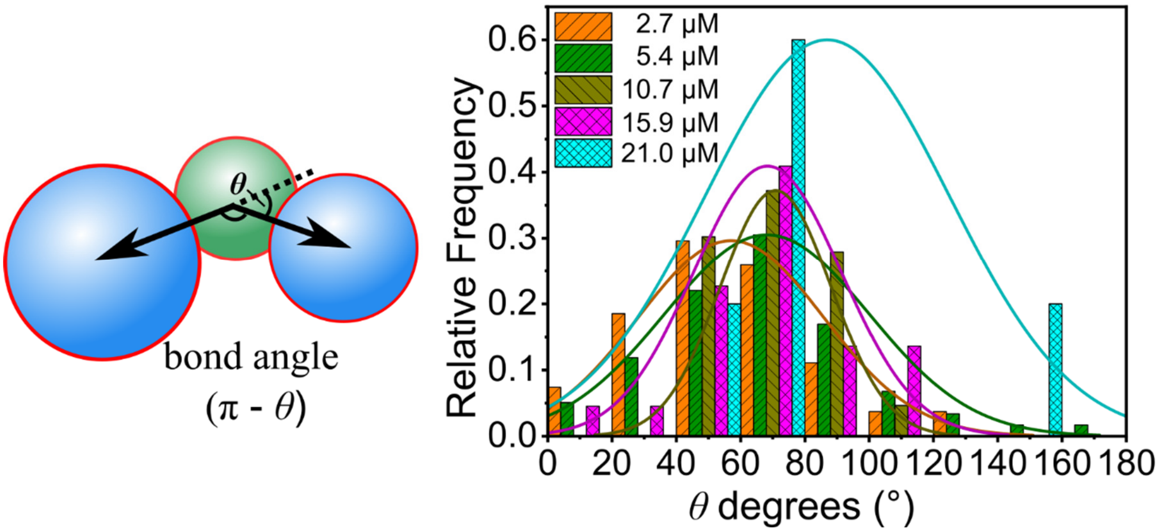

Figure S10: A schematic illustrating the θ degrees distribution between interlinked UTP/pLL and UTP/R_{10} droplets within the self-organized structure at different αSyn concentrations. **It should be noted that the data points for αSyn = 21.0 µM are insufficient due to the formation of a network structure.*

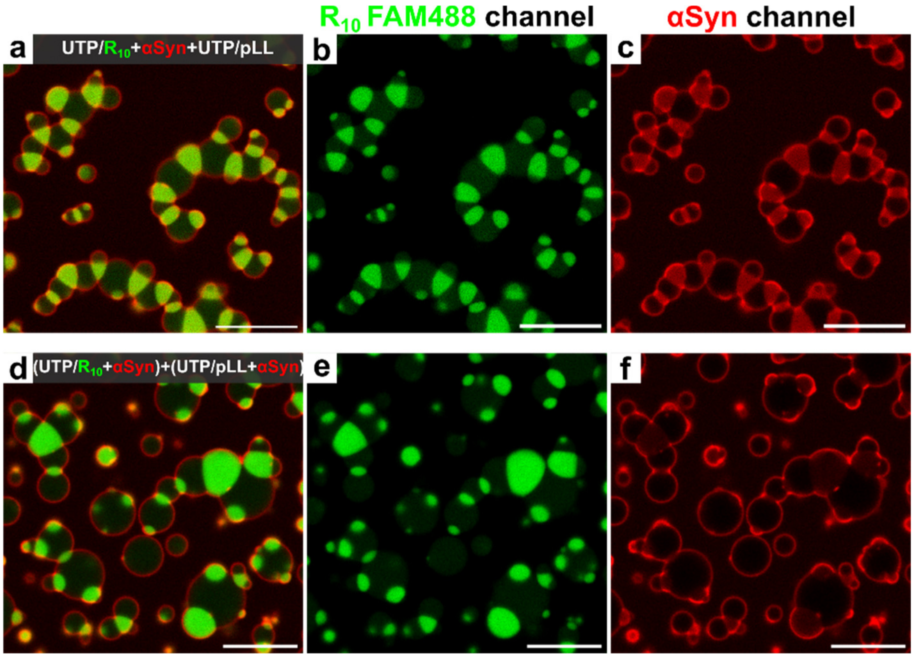

Figure S11. (a, d) Composite confocal images of different αSyn addition sequences into UTP/pLL/R_{10} coacervates at 1800s, showing a chain-like structure. (b,c,e,f) Separate channels of (a) and (d). The images show fluorescence from (b,e) FAM-Ahx-R_{10} (green, FAM488 channel) and (c,f) AF647-labeled αSyn (red, αSyn channel). All scale bars represent 10 μm.

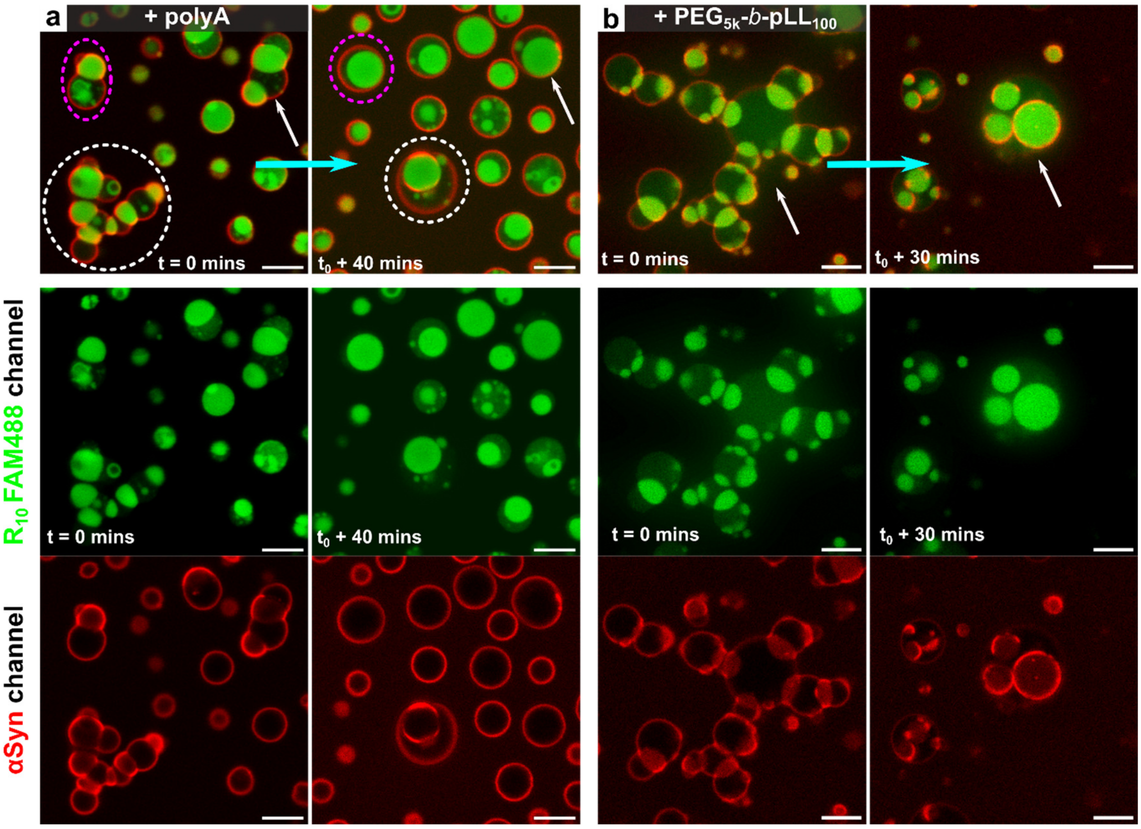

Figure S12. Composite images from confocal fluorescence microscopy showing the transformation of the chain-like structure after adding (a) negatively charged polyA (0.3 mg/mL) and (b) positively charged PEG_{5k}-*b*-pLL_{100} (2.5 mM), along with separate channels illustrating the transformation process of chain-like structures after adding polyA and PEG_{5k}-*b*-pLL_{100}. Fluorescence is shown from FAM-Ahx-R_{10} (green, FAM488 channel) and AF647-labeled αSyn (red, αSyn channel). All scale bars represent 5 μm.

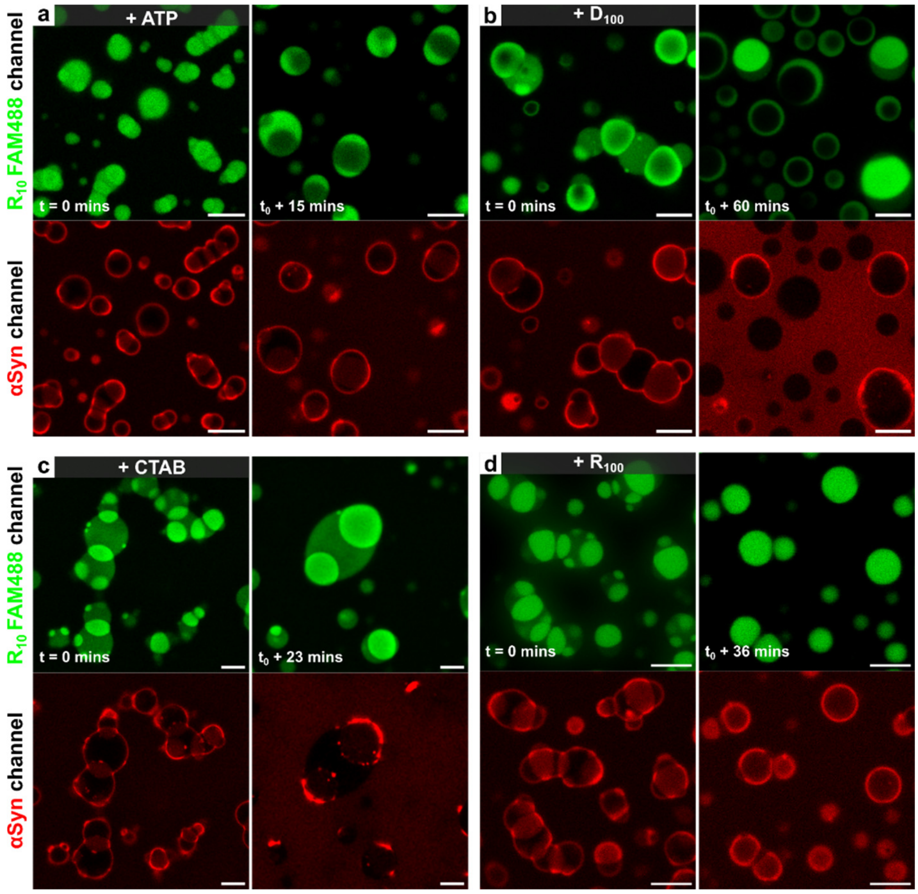

Figure S13. (a-d) Separate channels illustrating the transformation process of chain-like structures after adding different (a,b) negatively and (c,d) positively charged molecules. Fluorescence is shown from FAM-Ahx-R_{10} (green, FAM488 channel) and AF647-labeled αSyn (red, αSyn channel). All scale bars represent 5 µm.

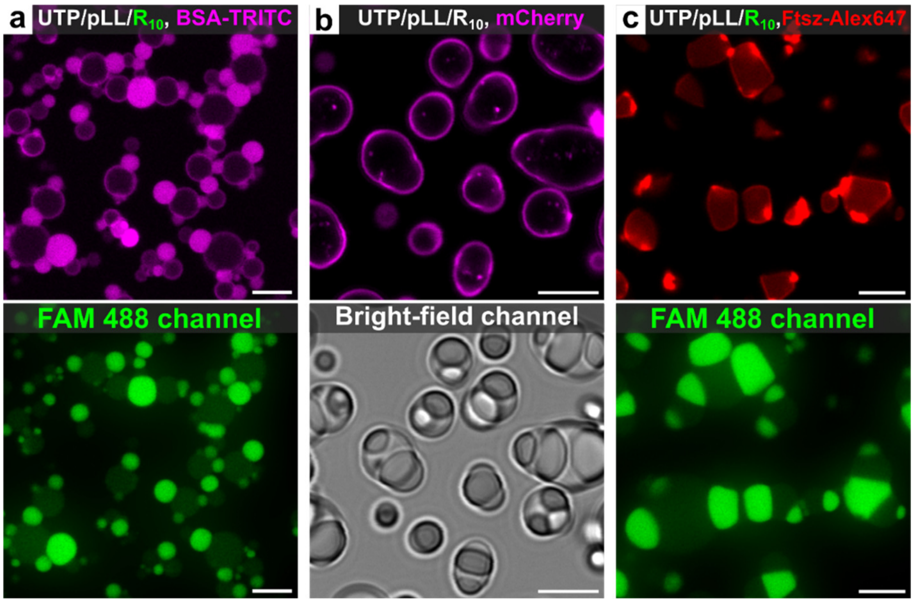

Figure S14: (a-c) Separate channels showing the addition of various negatively charged proteins at different concentrations to UTP/pLL/R_{10} multiphase coacervates: (a) BSA-TRITC at 2.5 µM; (b) mCherry at 0.36 µM; and (c) FtsZ at 2.0 µM, some FtsZ aggregates were observed. Fluorescence is shown from FAM-Ahx-R_{10} (green, FAM488 channel); BSA-TRITC and mCherry (magenta, mCherry channel); and AF647-labeled FtsZ (red, AF647channel). All scale bars represent 5 µm.

Various interfacial macromolecules regulate coacervate partial wetting and self-organization

BSA, mCherry, FtsZ, and PEG_{1k}-*b*-$pGlu_{100}$ were added at a comparable concentration to αSyn (2.7 µM) into the UTP/pLL/R_{10} multiphase coacervates. BSA-TRITC behaves similarly to αSyn, as illustrated in the dynamic process shown in Figure S15. The number of droplets (maximum 34) of the chain (self-organizing structure) and the corresponding chain length (107.2 µm), increases after adding BSA-TRITC compared to αSyn (Figure 3a). This indicates that at similar concentrations, BSA-TRITC interacts more strongly with UTP/pLL/R_{10}, likely due to its higher negative charge density. Even at lower concentrations, BSA-TRITC induces partial wetting and the release of core droplets, forming chain-like structures (Figure S16a). In contrast, mCherry induces partial wetting without forming extensive chain-like structures. This behavior can be attributed to mCherry attaching to the UTP/pLL interface without partitioning into the UTP/R_{10} coacervates. Additionally, the lower charge density and concentration of mCherry compared to αSyn and BSA-TRITC contribute to this phenomenon. Furthermore, at a relatively higher concentration of 0.71 µM, mCherry induces aggregation within the multiphase coacervate system, complicating the matching of concentration levels with the other two proteins (Figure S16b).

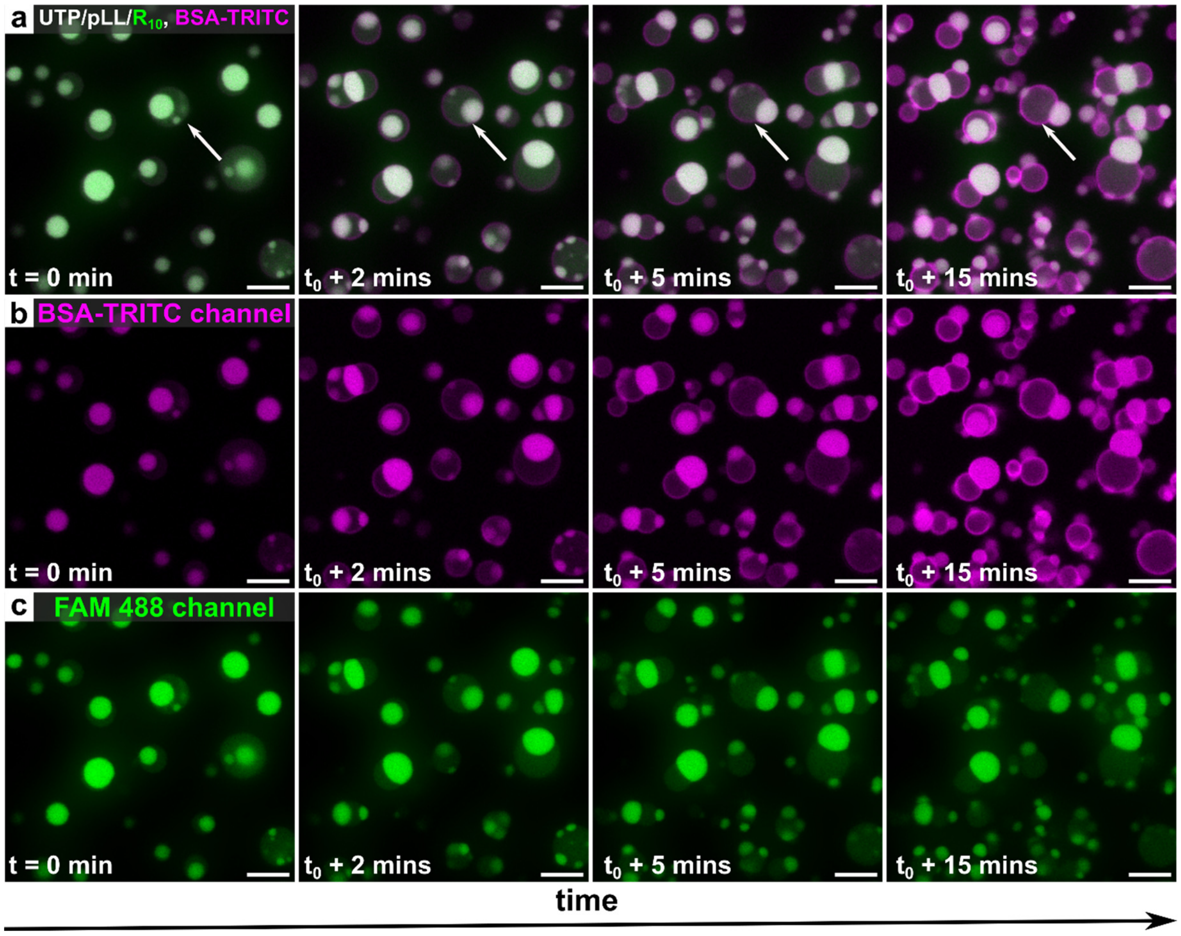

Figure S15: **Self-organization of multiphase coacervates.** Snapshots of composite and separate channel images from confocal fluorescence microscopy, illustrate the BSA-TRITC-induced partial wetting (indicated by the white arrows) and self-organization process of the UTP/pLL/R_{10} multiphase coacervates. Fluorescence is shown from BSA-TRITC (magenta) and FAM-Ahx-R_{10} (green). All scale bars represent 5 µm.

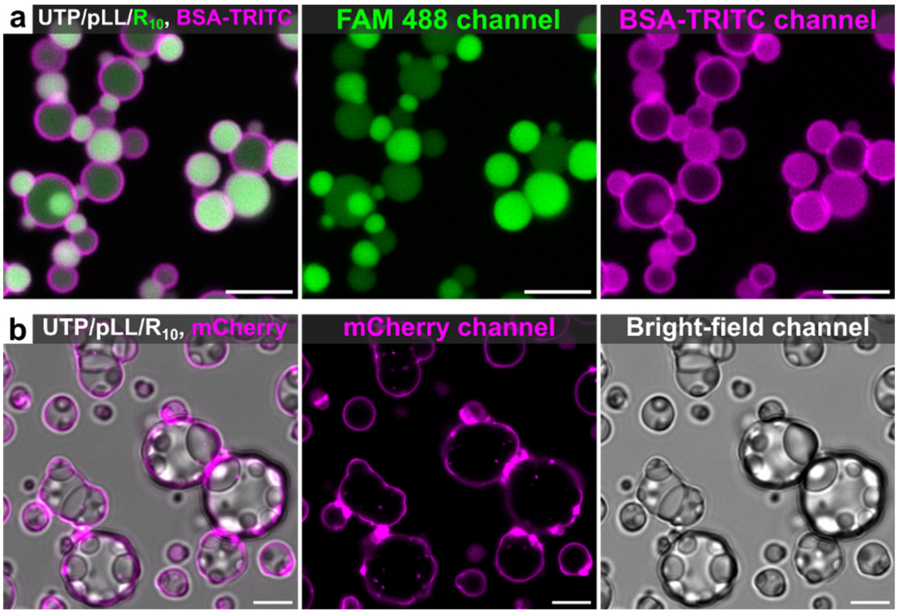

Figure S16: (a,b) Addition of different negatively charged proteins at different concentrations to the UTP/pLL/R_{10} multiphase coacervates: (a) BSA-TRITC at 2.0 µM, t = 40 mins; and (b) mCherry at 0.71 µM, t = 32 mins. Fluorescence images show BSA-TRITC and mCherry in magenta, and FAM-Ahx-R_{10} in green. All scale bars represent 5 µm.

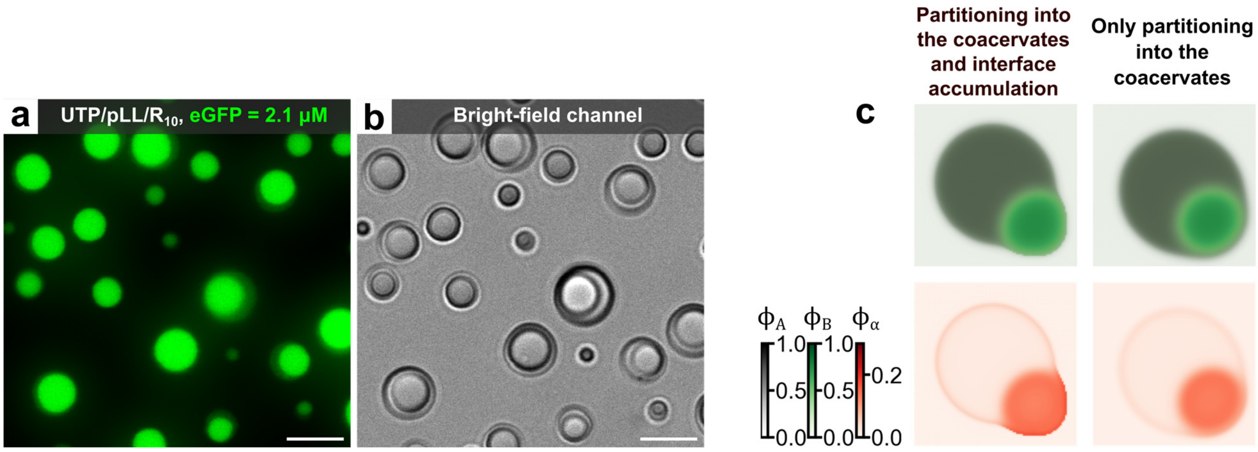

Figure S17: (a,b) Addition of eGFP to the UTP/pLL/R_{10} multiphase coacervates: (a) fluorescence confocal image and (b) bright-field image (t = 34 mins); (c) Comparison of partial wetting with and without interface accumulation for $\phi_{\alpha}^{(out)} = 0.01$. All scale bars represent 5 µm.

The stability of chain-like coacervate structures

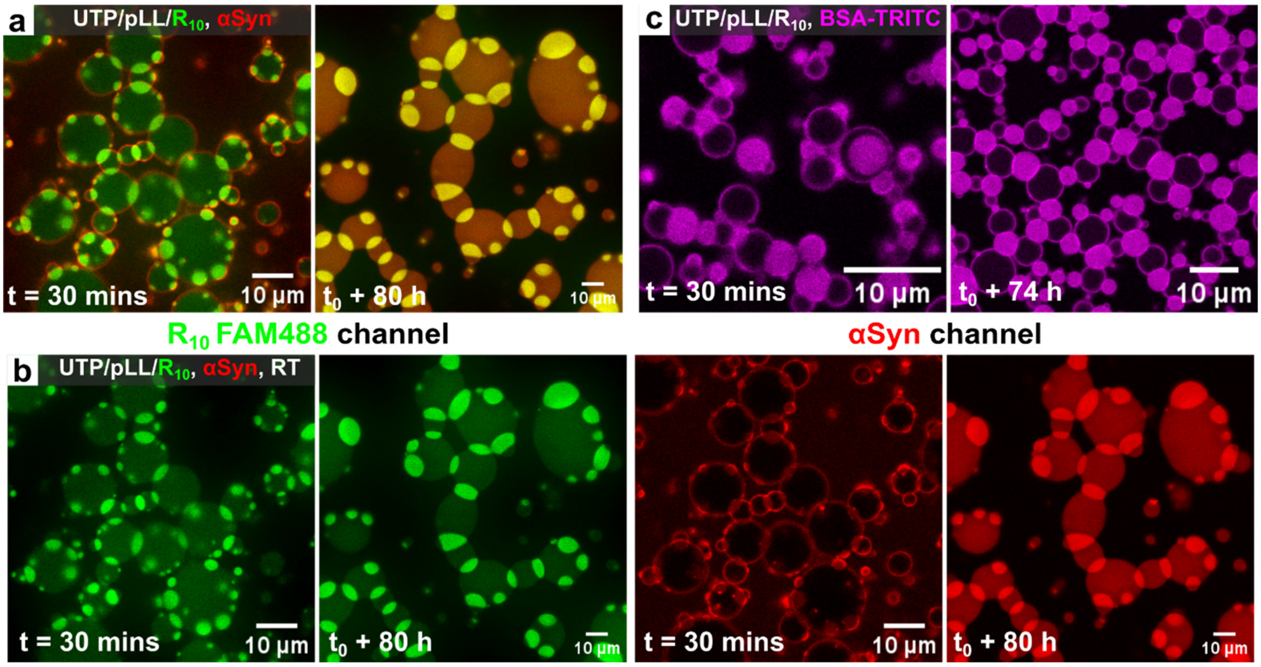

Figure S18. Composite and separate channel images from confocal fluorescence microscopy showing the stability study of chain-like structures at room temperatures with the addition of different proteins to the multiphase coacervates: (a,b) αSyn, and (c) BSA-TRITC. Fluorescence is shown from FAM-Ahx-R_{10} (green), TRITC labeled BSA (magenta), and AF647-labeled αSyn (red).

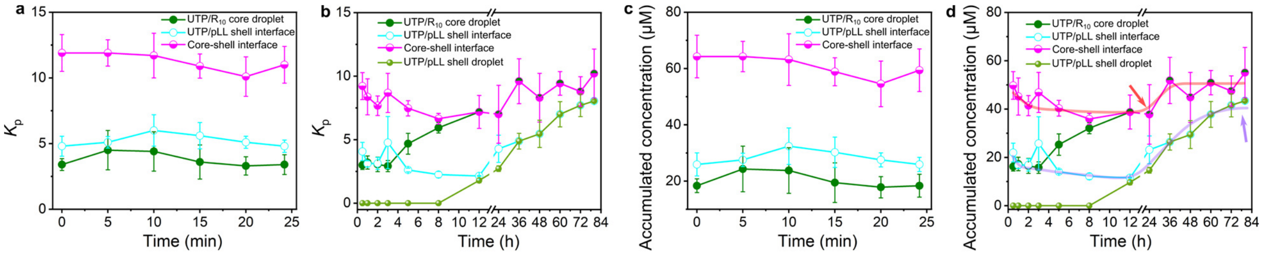

Figure S19. (a,b) Partitioning coefficient (K_p) and (c,d) accumulated concentration of αSyn in the UTP/R_{10} core, UTP/pLL shell, shell interface, and core–shell interface as a function of time. Panels (a) and (c) present a 24-minute time-lapse (cf. Figure 3a2) following the addition of αSyn to UTP/pLL/R_{10} multiphase coacervates, while panels (b) and (d) show an 80-hour time-lapse under identical conditions (cf. Figure S18). The red and purple curves and arrows are provided as visual guides.

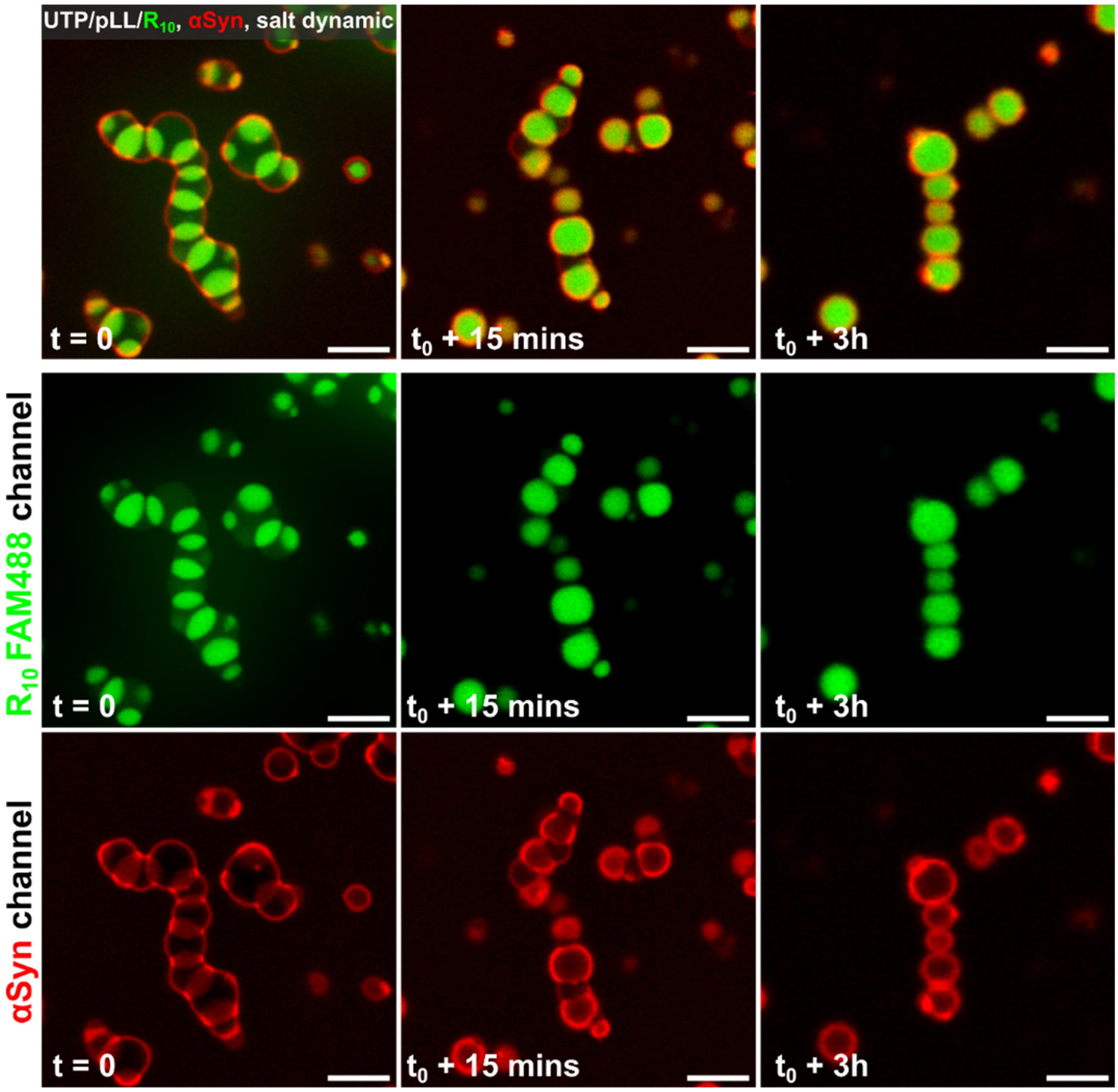

Figure S20. Composite and separate channel images from confocal fluorescence microscopy showing the stability study of chain-like structures at different salt concentrations at RT. Fluorescence is shown from FAM-Ahx-R_{10} (green) and AF647-labeled αSyn (red). All scale bars represent 5 μm.

2. Supplementary theory

Phase field model of multiphase coacervates

The free energy density of the multiphase coacervates is described by a Flory-Huggins model.

$$f(\phi_A, \phi_B, \phi_\alpha) = \frac{\phi_A}{n_A} ln\,\phi_A + \frac{\phi_B}{n_B} ln\,\phi_B + \frac{\phi_\alpha}{n_\alpha} ln\,\phi_\alpha$$
$$+ (1-\phi_A-\phi_B-\phi_\alpha)\,ln(1-\phi_A-\phi_B-\phi_\alpha) + \chi_{AB}\phi_A\phi_B + \chi_{A,\alpha}\phi_A\phi_\alpha + \chi_{B,\alpha}\phi_B\phi_\alpha$$
$$+ \chi_{A,sol}\phi_A(1-\phi_A-\phi_B-\phi_\alpha) + \chi_{B,sol}\phi_B(1-\phi_A-\phi_B-\phi_\alpha)$$
$$+ \chi_{\alpha,sol}\phi_\alpha(1-\phi_A-\phi_B-\phi_\alpha) \tag{S1}$$

with $\phi_A, \phi_B, \phi_\alpha$ the volume fraction of A, B and α,, n_A, n_B, n_α the ratio of the molecular volumes of A, B and α relative to the molecular volume ν, $\chi_{AB}, \chi_{A,\alpha}, \chi_{B,\alpha}, \chi_{A,sol}, \chi_{B,sol}, \chi_{\alpha,sol}$ are the Flory-Huggins interaction parameters. We note that the volume fraction of the solvent, ϕ_S, fulfils $\phi_S = 1 - \phi_A - \phi_B - \phi_\alpha$ and hence does not appear explicitly in Eq. (S1).

The dynamics of the multiphase system are described by

$$\frac{\partial \phi_A}{\partial \tilde{t}} = \tilde{\nabla}\left[\phi_A(1-\phi_A-\phi_B-\phi_\alpha)n_A\tilde{\nabla}\left[\frac{\partial f}{\partial \phi_A} - \widetilde{\kappa_A}\widetilde{\nabla^2}\phi_A - \widetilde{\sigma_A}\widetilde{\nabla^2}\phi_\alpha\right]\right] \tag{S2}$$

$$\frac{\partial \phi_B}{\partial \tilde{t}} = \tilde{\nabla}\left[\phi_B(1-\phi_A-\phi_B-\phi_\alpha)n_B\tilde{\nabla}\left[\frac{\partial f}{\partial \phi_B} - \widetilde{\kappa_B}\widetilde{\nabla^2}\phi_B - \widetilde{\sigma_B}\widetilde{\nabla^2}\phi_\alpha\right]\right] \tag{S3}$$

$$\frac{\partial \phi_\alpha}{\partial \tilde{t}} = \tilde{\nabla}\left[\phi_\alpha(1-\phi_A-\phi_B-\phi_\alpha)n_\alpha\tilde{\nabla}\left[\frac{\partial f}{\partial \phi_\alpha} - \widetilde{\kappa_\alpha}\widetilde{\nabla^2}\phi_\alpha - \widetilde{\sigma_A}\widetilde{\nabla^2}\phi_A - \widetilde{\sigma_B}\widetilde{\nabla^2}\phi_B\right]\right], \tag{S4}$$

where we use the dimensionless variables

$$\tilde{t} = \frac{t}{\tau}, \text{with } \tau = \frac{\nu^{3/2}}{\Lambda^{(0)}k_B T};\ \tilde{\nabla} = \nu^{-2/3}\nabla;\ \tilde{\kappa_i} = \kappa_i\nu^{-2/3}, \text{for } i = A, B, \alpha;\ \tilde{\sigma_i} = \sigma_i\nu^{-2/3}, \text{for } i = A, B. \tag{S5}$$

The following simulation parameters are used

$$n_A = 1;\ n_B = 1;\ n_\alpha = 2;\ \chi_{A,B} = 2.6;\ \chi_{A,\alpha} = 1;\ \chi_{B,\alpha} = -1;\ \chi_{A,sol} = 2.6;\ \chi_{B,sol} = 4.8;\ \chi_{A,\alpha} = 1;$$
$$\widetilde{\kappa_A} = 2;\ \widetilde{\kappa_B} = 4;\ \widetilde{\kappa_\alpha} = 4;\ \widetilde{\sigma_A} = -1.4;\ \widetilde{\sigma_B} = -3.4 \tag{S6}$$

We solve the dynamic equations using the spectral method, for a quadratic simulation box with boxlength $L = 50\nu^{1/3}$ and grid size $\Delta x = 50/96\ \nu^{1/3}$. To relax the initial conformation, which exhibits sharp edges, the first 1000 simulation steps are performed with a time step of $\Delta t = 5 \cdot 10^{-4}\tau$. The subsequent production run is performed with a time step of $\Delta t = 1 \cdot 10^{-3}\tau$. To generate a starting configuration, a value of ϕ_α in the dilute phase is set. The corresponding values of ϕ_A and ϕ_B for a three-phase equilibrium – or two-phase equilibrium for ϕ_α=0, are determined. The starting configuration is then composed of intersecting circles, where the value of ϕ_A, ϕ_B and ϕ_α in each circle and in the dilute surrounding is set to the respective equilibrium value. The simulation are performed for a total simulation time to 42075 τ for dimers and 69300 τ for the trimer. The start configurations for all simulations are shown in Figure S21.

Impact of interfacial accumulation on partial wetting

To test the importance of the accumulation of proteins at the interface, we set the parameters that promote interface accumulation to zero ($\widetilde{\sigma_A} = 0$; $\widetilde{\sigma_B} = 0$). We consider a system with $\phi_\alpha^{(out)} = 0.01$, i.e. the highest concentration of the alpha field, that we considered in the main text. We see the onset of partial wetting (Figure S17c), i.e. droplet B (green, core droplet) is not completely enclosed by droplet A (black, shell droplet). However, the small interface that droplet B forms with the dilute environment is not sufficient to form a distinct dimer structure and consequently coacervate polymers. We conclude that while partitioning of the α field into the B-rich phase alters the surface tension and the contact angle between the droplets, this effect is considerably weaker than in the case of interface accumulation.

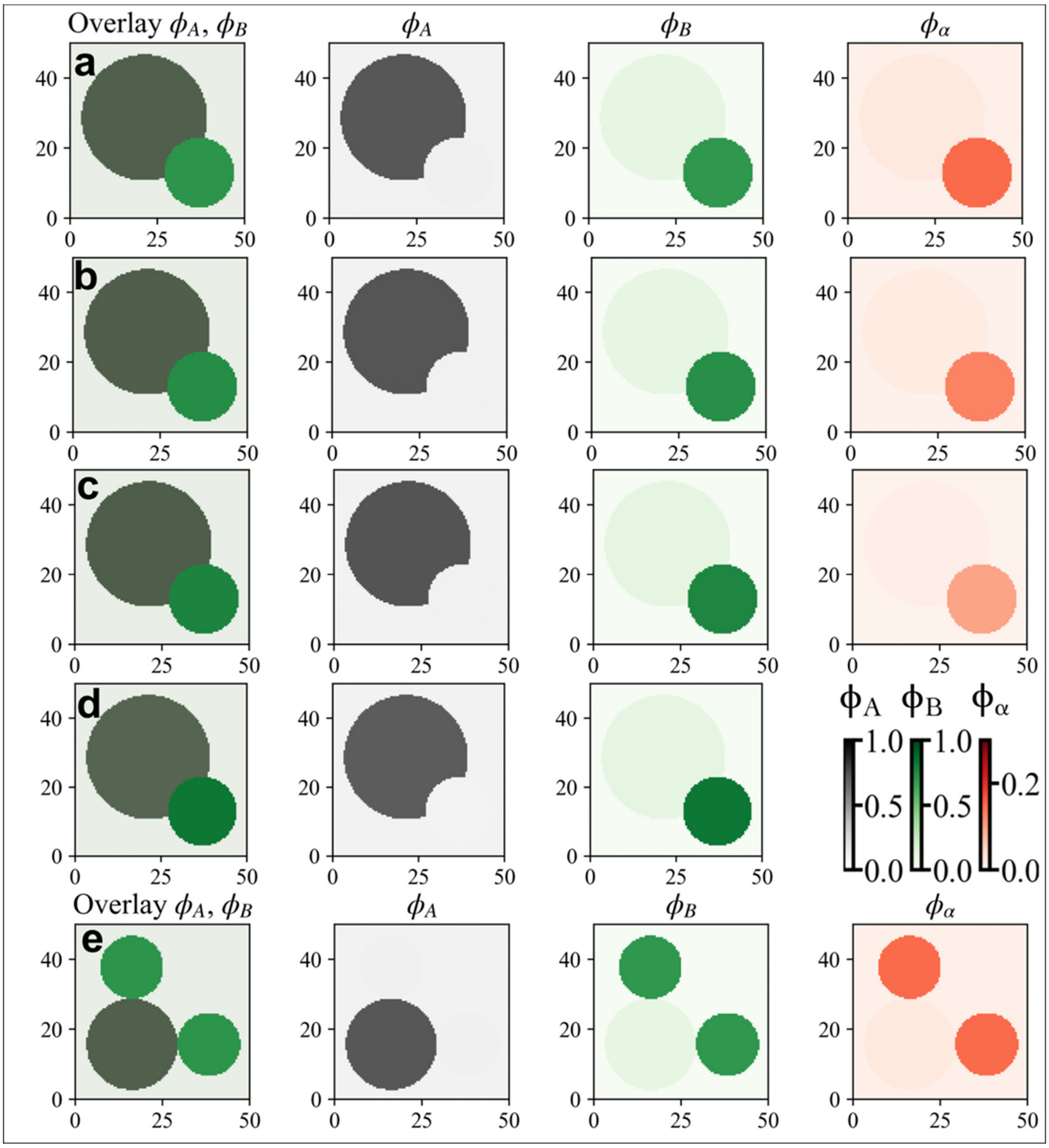

Figure S21. Start configuration of the numerical simulations: (a) $\phi_\alpha^{(out)} = 0.001$; (b) $\phi_\alpha^{(out)} = 0.005$; (c) $\phi_\alpha^{(out)} = 0.01$; (d) $\phi_\alpha^{(out)} = 0$; (e) $\phi_\alpha^{(out)} = 0.001$.

3. Supplementary Tables

Table S1. Critical salt concentrations (CSC) for coacervates formed by negatively charged molecules (UTP, ATP, D_{100}, and polyA) with either pLL or R_{10}, as well as UTP with R_{100}. Turbidity measurements were performed in triplo and error represents the standard deviation (n = 3). All samples were captured in brightfield view.

Coacervate	CSC ± Std (mM)	Microscopy	Coacervate	CSC ± Std (mM)	Microscopy
**UTP/pLL**	75.3 ± 0.8	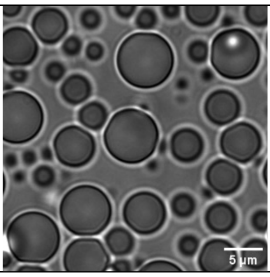	**UTP/R_{10}**	292.5 ± 5.0	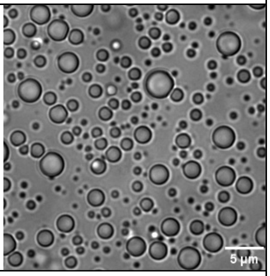
**UTP/R_{100}**	861.0 ± 17.5	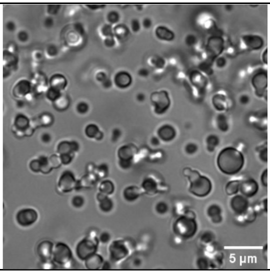			
**ATP/pLL**	400.6 ± 3.3	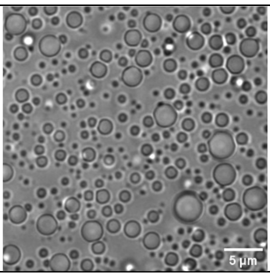	**ATP/R_{10}**	527.1 ± 11.1	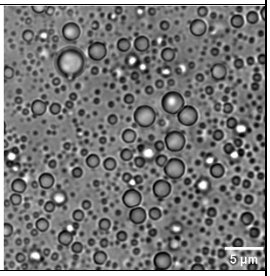
**D_{100}/pLL**	1090.3 ± 5.3	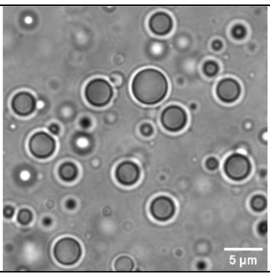	**D_{100}/R_{10}**	1509.3 ± 37.3	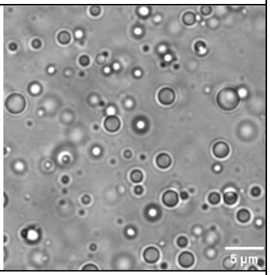
**polyA/pLL**	2758.5 ± 163.9	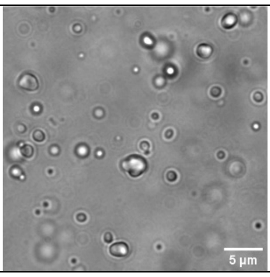	**polyA/R_{10}**	> 3000.0	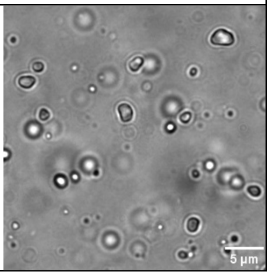

Table S2. Summary of the maximum number of droplets per chain, chain length, partitioning coefficient (K_p), and accumulated concentration of interfacial proteins within the UTP/R_{10} core, UTP/pLL shell, and the core–shell interface. The table also includes contact angles between core and shell droplets and the interfacial tension ratio following the addition of various interfacial macromolecules to UTP/pLL/R_{10} multiphase coacervates (*cf.* Figure 3a1 and Figure 6). K_p and accumulated concentration values are not available (n.a.) for PEG_{1k}-b-pE_{100}, as it was not fluorescently labeled.

Interfacial macromolecules	mCherry (0.36 μM)	αSyn (2.7 μM)	Ftsz-AF647 (2.0 μM)	PEG_{1k}-*b*-pE_{100} (3.0 μM)	BSA-TRITC (2.5 μM)
Maximum droplets number per chain	6	16	20	28	34
Chain length (μm)	11.6	22	44.7	58.3	107.2
K_{p1} (UTP/R_{10} core droplet)	9.8 ± 2.5	2.7 ± 0.5	209.7 ± 36.4	n.a.	19.4 ± 1.7
K_{p2_int} (UTP/pLL)	45.5 ±7.1	6.9 ± 1.7	11.2 ± 3.2	n.a.	11.1 ± 2.3
K_{p3_int} (core-shell interface)	80.3 ± 16.7	19.7 ± 2.6	418.7 ± 69.2	n.a.	19.4 ± 1.7
K_{p3}/ K_{p2_int}	1.8	2.9	37.4	n.a.	1.7
K_{p3_int} /K_{p1}	8.2	7.3	2	n.a.	1
K_{p2_int}/K_{p1}	4.6	2.6	0.05	n.a.	0.6
$C_{\text{UTP/R10 core droplet}}$	3.5	7.3	419.4	n.a.	48.5
$C_{\text{UTP/pLL shell interface}}$	16.4	18.6	22.4	n.a.	27.8
$C_{\text{core-shell interface}}$	28.9	53.2	837.4	n.a.	48.5
$2\pi - \beta_{UTP/R_{10}} - \beta_{UTP/pLL}$ (Contact angle) (°)	146.2 ± 9.1	111.4 ± 15.7	106.4 ± 15.7	125.2 ± 11.5	78.4 ± 19.5
γ_1/γ_2	5.7 ± 3.5	2.0 ± 0.9	1.1 ± 0.1	1.0 ± 0.2	3.5 ± 0.6

4. Supplementary movie captions

Movie S1. This video recorded the dynamic process of adding AF647-labeled αSyn into the multiphase UTP/pLL/R_{10} coacervate droplets. The AF647-labeled αSyn molecules interact with the multiphase coacervate interfaces, inducing partial wetting and triggering the release of the inner droplets. In this video, the replay speed is 50 times faster than the recorded experiment (a time stamp is shown at the top left, the total time is 5 min) (Figure 1d).

Movie S2. This video recorded the dynamic self-organization process of UTP/pLL and UTP/R_{10} coacervates following the addition of AF647-labeled αSyn to the multiphase UTP/pLL/R_{10} coacervate droplets. In this video, the replay speed is 100 times faster than the recorded experiment (a time stamp is shown at the top left, the total time is 19 min 10s) (Figure 2).

Movie S3. This video shows the introduction of negatively charged polymer D_{100} into self-organized structures, resulting in the removal of αSyn from the coacervate droplet interfaces and the subsequent reversion of the self-organized structure to a multiphase coacervate state. The video is presented at a playback speed 360 times faster than the actual experiment, with a timestamp displayed in the top left corner, representing a total experimental duration of 60 min (Figure 5c).
